# Preliminary Assessment of Anticancer Activity of Aqueous Meadowsweet (*Filipendula ulmaria* (L.) Maxim.) Extract in LoVo Colorectal Cancer Cells

**DOI:** 10.3390/biomedicines14071551

**Published:** 2026-07-10

**Authors:** Łukasz Sobczak, Agata Wszołek, Wojciech Żwierełło, Kinga Rybak, Anna Nowakowska, Edyta Stępień-Zawal, Marcin Wilhelm, Magdalena Rutkowska, Dominika Ciosek, Katarzyna Marzoch, Izabela Gutowska, Agnieszka Maruszewska

**Affiliations:** 1 Institute of Biology, University of Szczecin, Felczaka 3c St., 70-453 Szczecin, Polandkinga.rybak@pum.edu.pl (K.R.); anna.nowakowska@usz.edu.pl (A.N.);; 2 Molecular Biology and Biotechnology Center, Institute of Biology, University of Szczecin, Wąska 13 St., 71-415 Szczecin, Poland; 3 Department of Medical Chemistry, Pomeranian Medical University in Szczecin, Powstańców Wlkp. 71 St., 70-111 Szczecin, Poland; wojciech.zwierello@pum.edu.pl (W.Ż.); izabela.gutowska@pum.edu.pl (I.G.); 4 Institute of Marine and Environmental Sciences, University of Szczecin, Adama Mickiewicza 16 St., 70-383 Szczecin, Poland; edyta.stepien-zawal@usz.edu.pl (E.S.-Z.); marcin.wilhelm@usz.edu.pl (M.W.)

**Keywords:** *Filipendula ulmaria* (L.) Maxim., plant extract, LoVo colon cancer cells, anticancer, apoptosis, phytotherapy

## Abstract

**Background/Objectives**: *Filipendula ulmaria* (L.) Maxim. (meadowsweet) is a medicinal plant traditionally used for its antioxidant and anti-inflammatory effects. There is also some data indicating its anticancer potential; however, its impact on colorectal cancer cells remains poorly understood. Here we investigated the cytotoxic and pro-apoptotic effects of an aqueous *F. ulmaria* extract on human LoVo colorectal cancer cells and analyzed some of the mechanisms underlying it. **Methods**: LoVo colorectal cancer cells were treated with the aqueous extract and analyzed for intracellular reactive oxygen species (ROS), mitochondrial membrane potential, DNA damage, lysosomal alterations, apoptosis-related mechanisms, and antioxidant activity. Phytochemical profiling was performed by HPLC-TOF/MS. **Results**: The extract elevated intracellular ROS levels, disrupted mitochondrial membrane potential, and induced DNA damage in LoVo cells. Activation of crucial caspases, along with increased p53 levels, confirmed engagement of both extrinsic and intrinsic apoptotic pathways. Changes in lysosomal fluorescence were also observed, indicating alterations in lysosomal properties. In chemical assays (FRAP, TAC, DPPH, ABTS, and superoxide scavenging), the extract demonstrated robust antioxidant capacity comparable to or exceeding that of ascorbic acid. Phytochemical profiling by HPLC-TOF/MS revealed a rich presence of bioactive flavonoids, phenolic acids, and coumarins. Altogether, our findings indicate that the extract’s cytotoxicity against colon cancer cells arises from a multifaceted mechanism involving oxidative stress, organelle dysfunction, and apoptosis induction. **Conclusions:** These results highlight *F. ulmaria* aqueous extract as a promising candidate for colorectal cancer phytotherapy as a form of supportive treatment and warrant further preclinical validation.

## 1. Introduction

Cancer has become one of the leading causes of death worldwide. In 2022, nearly 20 million new cancer cases were reported worldwide, and there were nearly 10 million cancer-related deaths [1].

This serious clinical problem requires the search not only for new treatment strategies, but also for new substances with anticancer potential. Throughout history, many medicines originated from plants, and modern pharmacognosy continues to explore plant-derived substances for therapeutic potential. Traditional herbal medicine systems, such as Chinese medicine (dating back nearly 3000 BCE) and Indian Ayurveda, have long utilized a vast array of plant species for healing [2,3]. Phytotherapy is nowadays defined as the use of plant-derived medications to prevent and treat disease [4]. Medicinal plants are those containing pharmacologically active compounds [5], and preparations made from them (raw materials, extracts, or herbal formulations) are known as herbal medicines [6]. Meadowsweet (*Filipendula ulmaria* (L.) Maxim.) contains numerous bioactive constituents, including phenolic glycosides (e.g., spiraeoside, spirein, salicin), flavonoids such as hyperoside and avicularin, tannins, and essential oils (e.g., vanillin, methyl salicylate) [7]. These compounds contribute to its therapeutic properties: flavonoids confer diaphoretic, diuretic, and cholagogic effects; salicylates provide anti-inflammatory and analgesic actions; and tannins impart astringent and antibacterial activity. Meadowsweet preparations have been used as antipyretic and diaphoretic remedies and as adjuncts for colds, urinary tract infections, and relief of joint and muscle pain [8,9,10,11,12]. Despite meadowsweet’s known medicinal uses, its effects on cancer cells—especially colorectal cancer cells—remain insufficiently explored. Our study addresses this gap by examining whether an aqueous extract of *F. ulmaria* can induce cytotoxic and pro-apoptotic effects in colorectal cancer cells, and by investigating the associated roles of oxidative stress and apoptosis. By clarifying meadowsweet’s mechanism of action against colorectal cancer cells, we aim to establish a basis for future research into its potential as a complementary anticancer therapy.

## 2. Materials and Methods

### 2.1. Preparation of Plant Extracts

Meadowsweet (*Filipendula ulmaria* (L.) Maxim.) stems were collected from wild populations in West Pomerania, Poland. The plant material was taxonomically identified, and a voucher specimen (ID: 19/16) was deposited in the Institute of Biology, University of Szczecin. An aqueous extract was prepared by extracting 10 g of dried, ground plant material in 100 mL of deionized water at 80 °C for 3 h with continuous stirring. The mixture was then filtered, and the filtrate was lyophilized. The resulting dry extract was stored at −80 °C until use.

### 2.2. Determination of Total Polyphenol Content in the Extract

Total polyphenol content was determined using the Folin-Ciocalteu colorimetric method. Briefly, 0.5 mL of appropriately diluted extract was mixed with 2.5 mL of 10-fold diluted Folin-Ciocalteu reagent (Sigma-Aldrich, Saint Louis, MO, USA). After 5 min. of incubation, 2 mL of 7.5% Na_2_CO_3_ (Sigma-Aldrich, Saint Louis, MO, USA) was added. The mixture was incubated at RT in the dark for 30 min., and absorbance was measured at 765 nm. Total polyphenols were quantified against a gallic acid (Sigma-Aldrich, Saint Louis, MO, USA) standard curve and expressed as milligrams of gallic acid equivalents (GAE) per gram of dry extract. All measurements were performed in triplicate.

### 2.3. Assessment of the Antioxidant Activity of the Extract

#### 2.3.1. Ferric Ion Reducing Antioxidant Power (FRAP) Assay

The method is based on the determination of the ability to reduce Fe^3+^ ions to Fe^2+^. The analysis involves spectrophotometric measurement of the reduction of the iron-2,4,6-tripyridyl-S-thiazine complex under the influence of an antioxidant compound. This is associated with the appearance of a blue color in the reaction mixture (A_max_ at λ = 700 nm). Ascorbic acid (Sigma-Aldrich, Saint Louis, MO, USA) was used as a standard. Standard and extract solutions were prepared for the assay at concentrations of 0.01, 0.05, 0.1, 0.5, and 1 mg/mL. Antioxidant activity was expressed as the degree of Fe^3+^ reduction (% of control). The assay was performed in at least three independent replicates.

#### 2.3.2. Total Antioxidant Capacity (TAC)—Molybdenum Blue Method

The TAC assay is based on the reduction of Mo^6+^ to Mo^5+^ (molybdenum blue) by antioxidants under acidic conditions, resulting in a green complex (A_max_ at λ = 695 nm). Ascorbic acid served as the standard. The extract and ascorbic acid were tested at 0.1, 0.25, 0.5, 0.75, 1, and 2 mg/mL. Antioxidant capacity was expressed as the percentage of Mo^6+^ reduced (relative to control). Each determination was carried out in at least three independent repeats.

#### 2.3.3. 2,2-Diphenyl-1-picrylhydrazyl (DPPH) Radical Scavenging Assay

2,2-diphenyl-1-picrylhydrazyl (DPPH) (Sigma-Aldrich, Saint Louis, MO, USA), whose solution has a dark violet color with A_max_ at λ = 517 nm, forms a stable radical. In reaction with a substance with antioxidant properties, it undergoes reduction, leading to the loss of color in the reaction mixture. The decrease in absorbance is proportional to the amount of the oxidized form of DPPH appearing and remaining in the solution. As a standard, ascorbic acid was used. The solutions of the standard and the extract were prepared at concentrations of 0.1, 0.5, 1, 10, and 50 mg/mL. The antioxidant activity was expressed as the percentage of DPPH radical scavenging. Each measurement was performed in at least three independent replicates.

#### 2.3.4. Determination of Antioxidant Activity Using 2,2′-Azino-bis(3-ethylbenzothiazoline-6-sulfonic Acid) (ABTS) Radical Cation Decolorization Assay

Antioxidant capacity was further evaluated using the ABTS radical cation decolorization assay. The ABTS^•+^ radical cation was generated by reacting 7 mM ABTS stock solution (Sigma-Aldrich, Saint Louis, MO, USA) with 2.45 mM potassium persulfate (Sigma-Aldrich, Saint Louis, MO, USA) and incubating in the dark for 12–16 h. The resulting ABTS^•+^ solution was diluted with ethanol to an absorbance of 0.70 ± 0.02 at 734 nm. Extract and ascorbic acid solutions (reference standard) were prepared in ethanol at 0.05, 0.1, 0.25, 0.5, and 1.0 mg/mL. For each test, 1 mL of the diluted ABTS^•+^ solution was mixed with 10 μL of sample or standard. After 6 min of incubation at room temperature, absorbance at 734 nm was recorded. The percentage of ABTS^•+^ scavenged was calculated relative to the. All experiments were conducted with at least three independent replicates.

#### 2.3.5. Superoxide Anion Radical Scavenging

The ability of the extract to scavenge superoxide anion radicals (O_2_^•−^) was assessed by measuring its inhibition of nitroblue tetrazolium (NBT, (Sigma-Aldrich, Saint Louis, MO, USA)) reduction. Superoxide radicals were generated by illuminating a reaction mixture containing riboflavin (20 μM) (Pol-Aura, Dywity, Poland), methionine (13 mM) (Pol-Aura, Dywity, Poland), NBT (75 μM), and EDTA (Sigma-Aldrich, Saint Louis, MO, USA) (0.1 mM) in 50 mM phosphate buffer (pH 7.8) with UV-C light (312 nm) for 10 min. Extract samples and ascorbic acid (standard) were prepared at 0.01, 0.1, 0.5, 1, and 10 mg/mL. For each trial, 1 mL of sample or standard was added to 2 mL of the reaction mixture. After the UV-C exposure, absorbance was read at 560 nm. The percentage inhibition of superoxide-induced NBT reduction was calculated relative to a control (reaction mixture without antioxidant). Each measurement was performed in at least three independent replicates.

### 2.4. Sample Preparation and HPLC-TOF/MS Analysis

For phytochemical profiling, 100 mg of the lyophilized extract powder was suspended in 900 µL of water/methanol (1:1, *v*/*v*). The sample was extracted on a ThermoMixer (Eppendorf, Hamburg, Germany) at 4 °C and 2000 rpm for 30 min, then centrifuged at 12,000× *g* for 10 min. (4 °C). The supernatant (400 µL) was collected and placed into a 2 mL autosampler vial for analysis. HPLC-TOF/MS analysis was performed using an Agilent 1260 HPLC system (Agilent Technologies, Santa Clara, CA, USA) coupled to an Agilent 6224 time-of-flight mass spectrometer (Agilent Technologies, Santa Clara, CA, USA) with an electrospray ionization (ESI) source. Chromatographic separation was achieved on a Waters ACQUITY BEH C18 column (Waters Corporation, Milford, MA, USA) (150 mm × 2.1 mm, 1.7 µm particle size) with a mobile phase of 0.1% formic acid in water (solvent A) and 0.1% formic acid in acetonitrile (Sigma-Aldrich, Saint Louis, MO, USA) (solvent B) at 0.30 mL/min. The gradient program ramped from 2% to 98% B over 35 min, held at 98% B for 5 min, then re-equilibrated to initial conditions (total run time 40 min). The injection volume was 2 µL. MS data were acquired in scan mode from *m*/*z* 50–900 in positive and negative modes. Key MS parameters included: drying gas flow 11 L/min, nebulizer pressure 50 psi, drying gas temperature 350 °C, and capillary voltage 3500 V. Metabolites were identified by comparing acquired spectra to an MS/MS spectral database. Detected compounds and their characteristics (retention time, molecular formula, exact mass, mass error) are reported.

### 2.5. In Vitro Cell Cultures

Human LoVo colorectal adenocarcinoma cells (DSMZ, Braunschweig, Germany, ACC-350) and human BJ normal fibroblasts (ATCC, Manassas, VA, USA, CRL-2522) were cultured under standard conditions (37 °C, 5% CO_2_, 95% humidity). LoVo cells were maintained in F-12K nutrient mixture (Kaighn’s modification; Gibco, Thermo Fisher Scientific, Waltham, MA, USA) supplemented with L-glutamine, 10% (*v*/*v*) heat-inactivated fetal bovine serum (FBS) (Sartorius AG, Göttingen, Germany), and 100 U/mL penicillin + 100 µg/mL streptomycin (Capricorn Scientific, Ebsdorfergrund, Germany). BJ cells were maintained in Eagle’s Minimum Essential Medium (Sigma-Aldrich, Saint Louis, MO, USA) supplemented with 10% (*v*/*v*) heat-inactivated fetal bovine serum (FBS). To passage, cell monolayers were detached with 0.25% trypsin, centrifuged (400× *g*, 5 min., 21 °C), resuspended in fresh medium, and seeded into new vessels at 20,000 cells/cm^2^. Prior to all experiments, cells were allowed to adhere and grow for 24 h (lag phase). Depending on the experiment, cells were exposed to the extract for 24, 48, 72, or 96 h.

### 2.6. Determination of Cytotoxicity Using 3-(4,5-Dimethylthiazol-2-yl)-2,5-diphenyltetrazolium Bromide (MTT) Assay

Cytotoxicity was assessed using the MTT colorimetric assay. LoVo cells or BJ cells were seeded in 96-well plates at 5000 cells per well in 90 µL of culture medium. After the lag phase, 10 µL of the aqueous plant extract was added to wells to achieve final concentrations in the range of 0.1–2.5 mg/mL for LoVo cells and 0.3–20 mg/mL for BJ cells. Control wells received 10 µL of water (vehicle control). Additionally, blank wells containing medium plus extract (no cells) were included to account for any non-enzymatic MTT reduction. Cells were incubated with the extract for 72 h. After treatment, 20 µL of MTT (Sigma-Aldrich, Saint Louis, MO, USA) solution (2.5 mg/mL) was added to each well, and plates were incubated for 3 h. The medium was then removed, and 100 µL of DMSO (Sigma-Aldrich, Saint Louis, MO, USA) was added per well to dissolve the formazan crystals. Absorbance was measured at 555 nm using a microplate reader. Cell viability was calculated as a percentage of the untreated control after subtracting blank well absorbances. IC_50_ values (extract concentration causing 50% viability reduction) were determined. All experiments were performed in at least triplicate.

### 2.7. Evaluation of Cell Cycle Distribution Using Propidium Iodide (PI) DNA Staining Assay

Cell cycle distribution was analyzed using propidium iodide (PI) staining of DNA content, followed by flow cytometry. LoVo cells were treated with the extract at IC_50_ (0.34 mg/mL). UV-C-treated (70 mJ/cm^2^, 20 min.) cells served strictly as a methodological laboratory positive control to validate the technical performance of our assay setups and to benchmark classic apoptotic responses. After 24, 48, or 72 h, cells were harvested and washed with cold PBS. Cell pellets were fixed by slowly adding ice-cold 70% ethanol (−20°C) to a final volume of 400 µL and incubating at −20°C for at least 24 h. Fixed cells were then centrifuged, washed twice with PBS, and resuspended in 200 µL of PBS containing RNase A (100 µg/mL) (Sigma-Aldrich, Saint Louis, MO, USA) and PI (20 µg/mL) (Sigma-Aldrich, Saint Louis, MO, USA). After 30 min. of incubation at RT in the dark, samples were analyzed using flow cytometry (FACSCalibur, Becton Dickinson, Franklin Lakes, NJ, USA). The orange fluorescence (FL-2 channel) of 5000 events was recorded (585/42 BP, λex = 488 nm) to measure the DNA content. All experiments were performed in at least triplicate.

### 2.8. Evaluation of the Type of Induced Cell Death Using Annexin V–Fluorescein Isothiocyanate (FITC) and Propidium Iodide (PI) Assay

To distinguish between apoptotic and necrotic cell death, a dual-staining assay with Annexin V-FITC and PI was employed. LoVo cells were treated with the extract at IC_50_ (0.34 mg/mL) for 72 h. UV-C-treated (70 mJ/cm^2^, 20 min.) cells served strictly as a methodological laboratory positive control to validate the technical performance of our assay setups and to benchmark classic apoptotic responses. After treatment, cells were collected and washed twice with cold PBS. Staining was performed using a commercial FITC Annexin V Apoptosis Detection Kit (BD Pharmingen, San Diego, CA, USA) according to the manufacturer’s protocol. Cells were resuspended in 50 µL binding buffer, then incubated with 2.5 µL Annexin V-FITC and 2.5 µL PI for 15 min. in the dark at 25 °C. After adding 100 µL of binding buffer, samples were analyzed using, collecting 5000 events per sample. The green fluorescence (FL-1) corresponding to the FITC signal (530/30 BP, λex = 488 nm), and the orange fluorescence (FL-2) corresponding to the PI signal (585/42 BP, λex = 488 nm) were recorded. Data were processed with Flowing Software 2. Based on staining patterns, cells were classified as live (Annexin V^−^/PI^−^), early apoptotic (Annexin V^+^/PI^−^), late apoptotic (Annexin V^+^/PI^+^), or necrotic (Annexin V^−^/PI^+^). All determinations were performed in at least triplicate.

### 2.9. Evaluation of Intracellular Reactive Oxygen Species Using 2′,7′-Dichlorodihydrofluorescein Diacetate (2′,7′-DCFH-DA) Assay

Intracellular ROS levels were measured using the fluorescent probe 2′,7′-dichlorodihydrofluorescein diacetate (DCFH-DA) (Sigma-Aldrich, Saint Louis, MO, USA). LoVo cells were treated with the extract at IC_50_ (0.34 mg/mL). UV-C-treated (70 mJ/cm^2^, 20 min.) cells served strictly as a methodological laboratory positive control to validate the technical performance of our assay setups and to benchmark classic apoptotic responses. At 24, 48, and 72 h of incubation, cells were harvested and centrifuged. Cell pellets were washed twice with PBS, then resuspended in 200 µL PBS containing 1 µM DCFH-DA. After 30 min. of incubation at 37 °C in the dark, intracellular ROS-dependent fluorescence was analyzed using flow cytometry (FACSCalibur). The green fluorescence (FL-1) of 5000 events was recorded (530/30 BP, λex = 488 nm). Data were analyzed using Flowing Software 2. Results are presented as fold-change in ROS fluorescence intensity relative to the negative control. All experiments were performed in at least triplicate.

### 2.10. Assessment of DNA Damage by γ-H2AX Staining

DNA double-strand breaks were evaluated by staining for phosphorylated histone H2AX (γ-H2AX). LoVo cells were treated with the extract at IC_50_ (0.34 mg/mL) for 72 and 96 h. UV-C-treated (70 mJ/cm^2^, 20 min.) cells served strictly as a methodological laboratory positive control to validate the technical performance of our assay setups and to benchmark classic apoptotic responses. After treatment, cells were washed with cold PBS and fixed in 1% paraformaldehyde (Sigma-Aldrich, Saint Louis, MO, USA) (10 min., on ice). Fixed cells were then permeabilized with 0.1% Triton X-100 (Sigma-Aldrich, Saint Louis, MO, USA) in PBS for 15 min. Cells were incubated for 1 h with an Alexa Fluor 488-anti-γ-H2AX mAb (BD Pharmingen, San Diego, CA, USA) (10 µg per sample) in PBS/Triton, followed by washing. Subsequently, cells were stained with PI (20 µg/mL) in the presence of RNase A (100 µg/mL) for 30 min. Samples were analyzed using flow cytometry (FACSCalibur), collecting 5000 events. Green fluorescence for Alexa Fluor 488 signal (530/30 BP, λex = 488 nm), and orange fluorescence for PI signal (585/42 BP, λex = 488 nm) were recorded. The percentage of γ-H2AX-positive cells (cells with DNA damage) was calculated. All determinations were performed in at least triplicate.

### 2.11. Evaluation of Changes in Lysosomal Fluorescence Using LysoTracker Green DND-26 Fluorescent Probe

Lysosomal staining was assessed using LysoTracker Green DND-26, a fluorescent dye that accumulates in acidic lysosomal compartments. LoVo cells were treated with the extract at IC_50_ (0.34 mg/mL). UV-C-treated (70 mJ/cm^2^, 20 min.) cells served strictly as a methodological laboratory positive control to validate the technical performance of our assay setups and to benchmark classic apoptotic responses. At 24, 48, and 72 h, cells were collected and centrifuged. Pellets were washed twice with PBS and resuspended in 200 µL of PBS containing 100 nM LysoTracker Green (Thermofisher Scientific, Waltham, MA, USA). After 30 min. of incubation at 37 °C in the dark, fluorescence was measured by flow cytometry (FACSCalibur). The green fluorescence (FL-1) of 5000 events was recorded (530/30 BP, λex = 488 nm). Data were analyzed with Flowing Software 2. Relative lysosomal fluorescence intensity was quantified and expressed relative to control cells. All experiments were performed in at least triplicate.

### 2.12. Analysis of Changes in Mitochondrial Membrane Potential (ΔΨm)

Changes in mitochondrial membrane potential (ΔΨm) were examined using the JC-1 dye. LoVo cells were treated with the extract at IC_50_ (0.34 mg/mL) for 24, 48, or 72 h. UV-C-treated (70 mJ/cm^2^, 20 min.) cells served strictly as a methodological laboratory positive control to validate the technical performance of our assay setups and to benchmark classic apoptotic responses. Following treatment, cells were collected and washed twice with PBS. Cell pellets were resuspended in 500 µL of PBS containing 2 µM JC-1 (Sigma-Aldrich, Saint Louis, MO, USA) and incubated at 37 °C for 20 min. in the dark. Fluorescence was analyzed using flow cytometry (FACSCalibur) with 5000 events per sample. JC-1 aggregates with red fluorescence were detected in the FL-2 channel (585/42 BP, λex = 488 nm), and JC-1 monomers with green fluorescence in the FL-1 channel (530/30 nm BP, λex = 488 nm). A decrease in the red/green fluorescence ratio indicates loss of ΔΨm. Data were analyzed using Flowing Software 2. The percentage of cells with depolarized mitochondria (lower ΔΨm) was quantified for each sample. All determinations were performed in at least triplicate.

### 2.13. Determining the Level of p53 Protein

The intracellular level of p53 protein was measured by flow cytometry using an FITC-conjugated anti-p53 mAb. LoVo cells were treated with the extract at IC_50_ (0.34 mg/mL) for 72 h. UV-C-treated (70 mJ/cm^2^, 20 min.) cells served strictly as a methodological laboratory positive control to validate the technical performance of our assay setups and to benchmark classic apoptotic responses. After treatment, cells were collected and washed twice with PBS. Cells were fixed in 0.1% paraformaldehyde for 15 min. at RT, then permeabilized with 0.5% Tween-20 for 15 min. After washing, cells were incubated for 30 min. in 100 µL of staining buffer with FITC-anti p53-mAb (BD Pharmingen, San Diego, CA, USA) (5 µL per sample). Cells were then washed and resuspended in PBS for analysis. Flow cytometry was performed on a FACSCalibur, collecting 5000 events per sample (green fluorescence FL-1 channel, 530/30 BP, λex = 488 nm). Data were analyzed with Flowing Software 2. p53 levels are expressed as fold-change in fluorescence intensity relative to control. All determinations were performed in at least triplicate.

### 2.14. Assessment of Caspase Activation Using Fluorochrome-Labeled Inhibitors of Caspases (FLICA) Assay

Caspase activation was detected using fluorochrome-labeled inhibitors of caspases (FLICA). LoVo cells were seeded at 20,000 cells/cm^2^ and treated with the extract at IC_50_ (0.34 mg/mL). UV-C-treated (70 mJ/cm^2^, 20 min.) cells served strictly as a methodological laboratory positive control to validate the technical performance of our assay setups and to benchmark classic apoptotic responses. At 24, 48, and 72 h, cells were harvested and incubated with caspase-specific FLICA probes: DEVD-FMK–FITC for caspase-3, IETD-FMK–FITC for caspase-8, and LEHD-FMK–FITC for caspase-9 (Invitrogen, Thermo Fisher Scientific, Waltham, MA, USA), according to the manufacturer’s instructions. After labeling, cells were washed and analyzed using flow cytometry (green fluorescence FL-1 channel, 530/30 BP, λex = 488 nm). For each sample, 5000 events were collected. The percentage of cells with activated caspases was determined by comparing treated samples to controls. Caspase activation is reported as fold increase over the negative control. All determinations were performed in at least triplicate.

### 2.15. Quantification of Gene Expression Using Real-Time Quantitative Reverse Transcription PCR (RT-qPCR)

Gene expression changes were measured by RT-qPCR. Total RNA was extracted from LoVo cells using the GeneMATRIX Universal RNA MiniPrep kit (EurX, Gdańsk, Poland) following the manufacturer’s protocol. RNA concentration and purity were assessed using a NanoDrop 2000 spectrophotometer (Thermo Fisher Scientific, Waltham, MA, USA). cDNA was synthesized from 1 µg of RNA (using the NG dART RT Kit (EurX, Gdańsk, Poland), and 10 ng of resulting cDNA was used per qPCR reaction. Real-time PCR was performed on a Bio-Rad CFX96 (Bio-Rad Laboratories, Hercules, CA, USA) system with SYBR Green master mix(EurX, Gdańsk, Poland) in a 20 µL reaction. Specific primer pairs were used for target genes for caspase-8, caspase-9, and p53, as well as the reference gene for β2-microglobulin (B2M) for normalization. Primer sequences were as follows: *CASP8* forward 5′-GGAGGAGTTGTGTGGGGTAA-3′, reverse 5′-GATCAGACAGTATCCCCGAGG-3′; *CASP9* forward 5′-GACAGGCTCTTAGCAGCTTCC-3′, reverse 5′-CACAAGTCACTAGCCCTGGAC-3′; *TP53* forward 5′-TCAACAAGATGTTTTGCCAACTG-3′ (targeting human *TP53*), reverse 5′-ATGTGCTGTGACTGCTTGTAGATG-3′; *B2M* forward 5′-AATGCGGCATCTTCAAACCTA-3′, reverse 5′-TGACTTTGT CACAGCCCAAGA-3′. The PCR thermal cycling included initial denaturation (95 °C, 3 min) followed by 40 cycles of 95 °C for 10 s and 60 °C for 30 s. Relative gene expression was calculated using the 2^−ΔΔCt method, with *B2M* used as the reference gene for normalization. Results were expressed as 2^−ΔΔCt.

### 2.16. DNA Fragmentation Detection Using Terminal Deoxynucleotidyl Transferase dUTP Nick-End Labeling (TUNEL) Assay

Apoptotic DNA fragmentation was assessed using the TUNEL assay using a BrdU-Red UTP kit (Invitrogen Thermo Fisher Scientific, Waltham, MA, USA)). LoVo cells were treated with the extract at IC_50_ (0.34 mg/mL) for 72 and 96 h. UV-C-treated (70 mJ/cm^2^, 20 min.) cells served strictly as a methodological laboratory positive control to validate the technical performance of our assay setups and to benchmark classic apoptotic responses. After treatment, cells were collected, washed with PBS, fixed in 1% paraformaldehyde, and then permeabilized in 70% ethanol (on ice). DNA strand breaks were labeled by terminal deoxynucleotidyl transferase (TdT) incorporation of BrdUTP at 3′-OH DNA ends, according to kit instructions. Cells were then stained with FITC-anti-BrdU mAb and counterstained with PI to label total DNA. Flow cytometry (FACSCalibur) was used to analyze 5000 events per sample. Green fluorescence for FITC signal (530/30 BP, λex = 488 nm) and orange fluorescence for PI signal (585/42 BP, λex = 488 nm) were recorded. TUNEL-positive cells (BrdU-incorporated, fragmented DNA) were quantified as a percentage of the total. All determinations were performed in at least triplicate.

### 2.17. Statistical Analysis

Quantitative data are presented as the mean or median (with dispersion measures such as standard deviation or quartiles as appropriate) of at least three independent experiments. Statistical analyses were performed using Statistica software (v13.3.721.1, TIBCO Software Inc., Palo Alto, CA, USA). Data normality was assessed with the Shapiro–Wilk test. Given that many datasets were non-parametric, group comparisons were typically made using the Mann–Whitney U test. A value of *p* ≤ 0.05 was considered statistically significant.

## 3. Results

**Figure 1:**
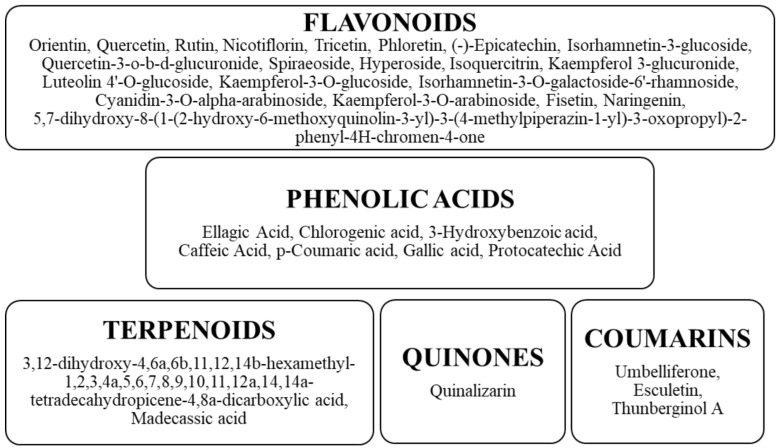
Chemical composition of secondary metabolites identified in the aqueous extract of *F. ulmaria* via HPLC-TOF/MS.

The total phenolic content of the aqueous *F. ulmaria* extract was found to be 230 ± 99 mg GAE per g of dry weight. Qualitative HPLC-TOF/MS analysis demonstrated a diverse array of secondary metabolites in the extract. A total of 34 different secondary metabolites were identified in the aqueous extract of *F. ulmaria* (Figure 1). Flavonoids were the predominant class, including quercetin derivatives (such as rutin and isoquercitrin) and kaempferol derivatives, alongside other compounds like (-)-epicatechin and naringenin. Several phenolic acids were also identified (e.g., gallic, ellagic, chlorogenic acids), as well as metabolites from other classes such as coumarins (e.g., umbelliferone) and terpenoids. This broad chemical profile suggests the extract has substantial biological activity potential. Figure 1 lists the major compounds detected. Supplementary Table S1 provides TIC chromatograms illustrating the extract’s metabolic profile, along with their retention times, formulas, and mass spectral data.

**Figure 2:**
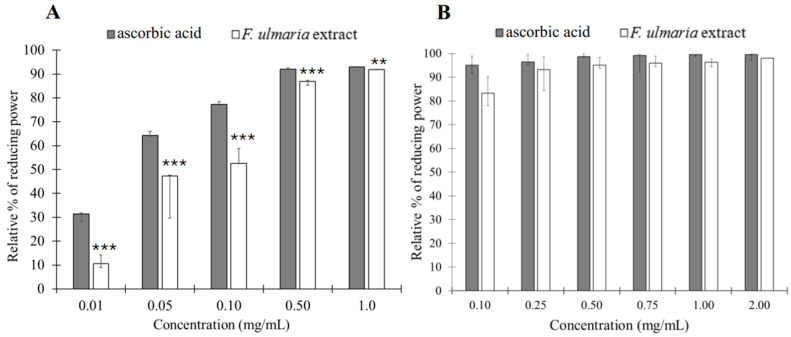
Assessment of the reducing power of the *F. ulmaria* extract using the (**A**) FRAP method and (**B**) TAC method. The level of significance of differences between the results for the tested sample (with the extract) and the reference sample (with ascorbic acid): ** *p* ≤ 0.01; *** *p* ≤ 0.001. The graphs depict medians and quartiles from at least three independent repetitions. Data obtained in antioxidant assays were collected in Table S2 in Supplementary Materials.

The aqueous *F. ulmaria* extract exhibited high antioxidant activity across multiple chemical assays. In the FRAP assay, the extract significantly reduced Fe^3+^ to Fe^2+^ ions at a level of about 50% at 0.05 and 0.1 mg/mL, and over 90%at 0.5 and 1 mg/mL, which was comparable to ascorbic acid (Figure 2A). Similarly, in the TAC assay, the extract reduced over 80% of Mo^6+^ to Mo^5+^, which was comparable to the reducing power of the reference antioxidant, ascorbic acid (Figure 2B).

**Figure 3:**
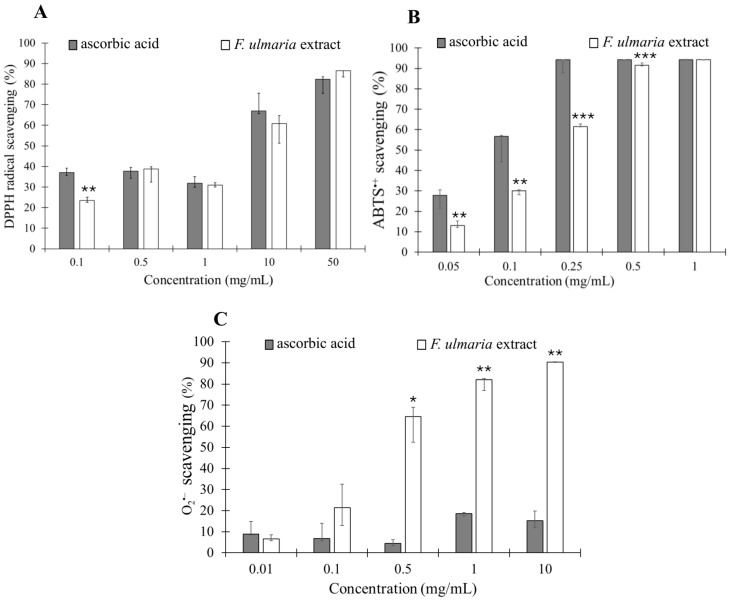
Assessment of the radical scavenging activity of the *F. ulmaria* extract using (**A**) DPPH method, (**B**) ABTS method, and (**C**) Superoxide anion radical scavenging assay. The level of significance of differences between the results for samples with the extract and samples with ascorbic acid: * *p* ≤ 0.05, ** *p* ≤ 0.01, *** *p* ≤ 0.001. The graphs depict medians and quartiles from at least three independent repetitions. Data obtained in antioxidant assays were collected in Table S2 in Supplementary Materials.

In free radical scavenging assays, the extract was also very effective. The percentage of DPPH radical scavenging for ascorbic acid and the *F. ulmaria* extract was similar at most concentrations used. Only at a concentration of 0.1 mg/mL was the activity for ascorbic acid approximately 40%, and for the tested extract approximately 25%. At concentrations of 0.5 and 1 mg/mL, the level of DPPH radical scavenging ranged from 30 to 40% for both samples, while at concentrations of 10 and 50 mg/mL, it ranged from 60% to almost 90% for both samples. (Figure 3A). In the ABTS assay, at the three lowest concentrations used, i.e., 0.05, 0.1, and 0.25 mg/mL, the standard samples with ascorbic acid demonstrated higher antioxidant potential, and the scavenging of the ABTS cation radical ranged from approximately 30% to over 90%, while for the tested extract, it ranged from approximately 15% to over 60%. At concentrations of 0.5 and 1 mg/mL, the results for the standard remained at a level of over 90% of the radical uptake capacity, while with increasing concentration, the antioxidant potential of the extract increased (Figure 3B). The percentage of O_2_^•−^ uptake at the lowest concentration used (0.01 mg/mL) was comparable for both tested substances and amounted to approximately 10%. At higher concentrations (0.5, 1, and 10 mg/mL), meadowsweet extract began to demonstrate significantly higher activity in neutralizing the superoxide anion radical—the radical uptake capacity ranged from 65 to 90%, while for ascorbic acid this activity was around 20%. (Figure 3C).

**Figure 4:**
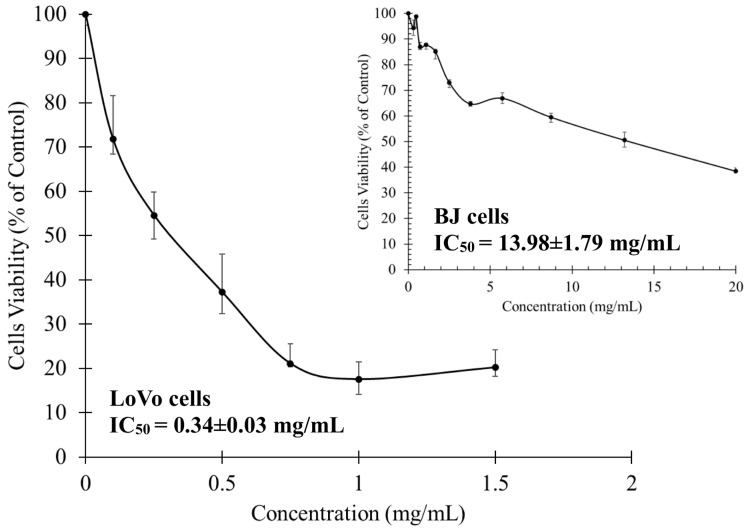
Cytotoxicity of the meadowsweet extract against LoVo cancer cell line and BJ cell line (normal fibroblasts). Cells were incubated in the presence of the tested extract in the range of 0.1–2.5 mg/mL for LoVo cells and 0.3–20 mg/mL for BJ cells for 72 h under the optimal conditions. The viability of the cell population was assessed using the MTT assay. The graph depicts medians and quartiles from at least three independent repetitions.

The extract also showed cytotoxic effects against LoVo cancer cells. In the MTT assay, treatment with the extract for 72 h significantly reduced cell viability in a dose-dependent manner. The mean IC_50_ (concentration causing 50% inhibition of viability compared to the control) was approximately 0.34 ± 0.03 mg/mL. Moreover, the extract was shown to have a weak effect on normal fibroblast BJ cells. The IC_50_ for these cells was as high as 14.03 mg/mL (Figure 4). Following the determination of this definitive 72 h IC_50_ value, this fixed concentration (0.34 mg/mL) was selected to systematically investigate the time-dependent downstream molecular mechanisms at earlier chronological intervals (24 h and 48 h) in the subsequent flow cytometry assays.

**Figure 5:**
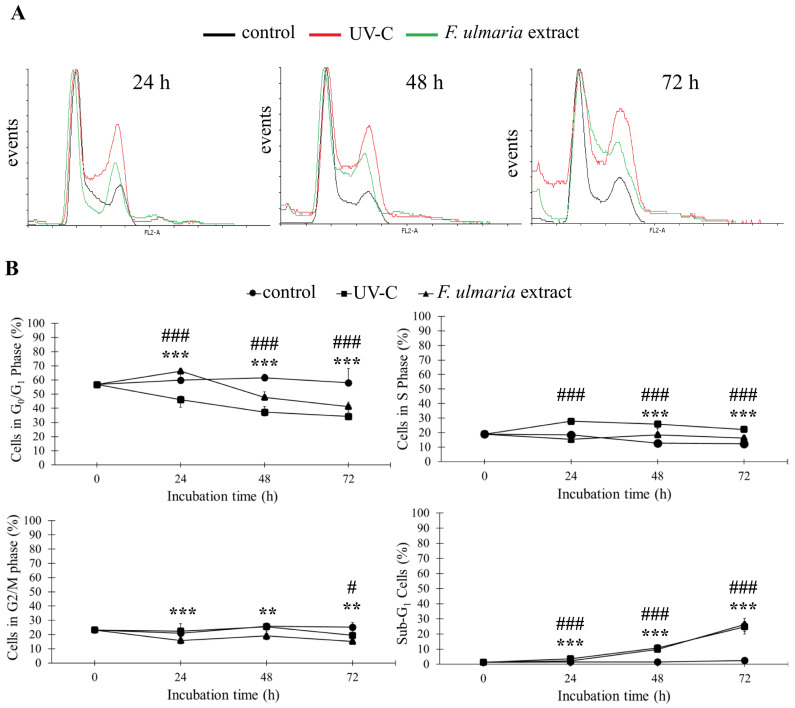
The effect of *F. ulmaria* extract on the distribution of LoVo cells in the cell cycle and the percentage of the sub-G1 fraction. (**A**) Representative histograms showing DNA content in control cells, UV-C-treated cells, and cells treated with *F. ulmaria* extract at 24 h, 48 h, and 72 h of incubation. (**B**) Analysis of the distribution of cells in the G_0_/G_1_, S, and G_2_/M phases of the cell cycle, as well as the percentage of the sub-G1 population. The graphs depict median values and quartiles from at least three independent replicates. The level of statistical significance of differences between the positive control sample (UV-C, #) or test sample incubated with the extract (*) compared to the negative control sample is represented as ** *p* ≤ 0.01, *** *p* ≤ 0.001; # *p* ≤ 0.05, ### *p* ≤ 0.001. The extract was consistently applied at a definitive concentration of 0.34 mg/mL.

Flow cytometric cell cycle analysis revealed that the extract did not arrest cells at a specific checkpoint (G_1_/S, or G_2_/M), but it did alter the distribution of cells among these phases (Figure 5). In the cell population incubated with the extract, a decrease in the percentage of cells in G_0_/G_1_ was observed at 48 and 72 h of treatment, reaching approximately 50% and 40%, respectively. The number of cells in the S and G_2_/M phases fluctuated slightly throughout the experiment, reaching 15%/16% [S/(G2/M)], 19%/19%, and 16%/15%, respectively, at the 24th, 48th, and 72nd hour of the experiment. The percentage of G_0_/G_1_-phase cells declined markedly after 72 h of exposure, coinciding with a significant rise in the sub-G_1_ population. The sub-G_1_ fraction represents cells with fractional DNA content, indicative of DNA fragmentation associated with cell death.

**Figure 6:**
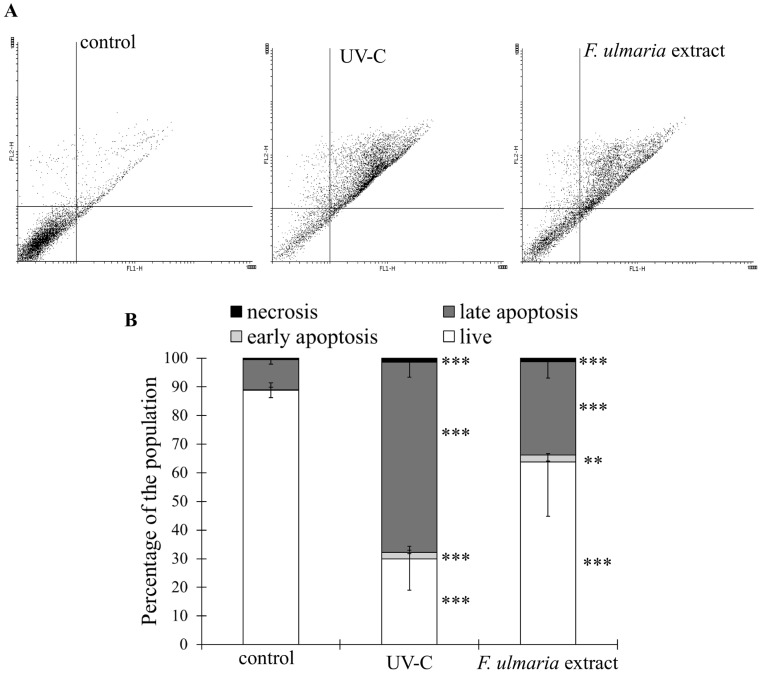
The effect of *F. ulmaria* extract on the induction of programmed cell death in LoVo cells. (**A**) Representative dot plots of LoVo cells from the negative control group, UV-C-treated group (positive control), and extract-treated group at 72 h of incubation. (**B**) Quantitative distribution of cells undergoing necrosis, early apoptosis, late apoptosis, and those remaining viable. The graph depicts median values and quartiles from at least three independent replicates. The level of statistical significance of differences between systems: the cells from the positive control (UV-C) or cells incubated with the extract, and the negative control cells: ** *p* ≤ 0.01; *** *p* ≤ 0.001. The extract was consistently applied at a definitive concentration of 0.34 mg/mL.

Consistently, an Annexin V/PI assay confirmed that apoptosis was the predominant mode of cell death induced by the extract (Figure 6). In the population of cells incubated with meadowsweet extract, live cells at 72 h of the experiment constituted approximately 64%, early apoptotic cells approximately 2%, late apoptotic cells over 32%, and necrotic cells, similar to early apoptotic cells, approximately 2%.

**Figure 7:**
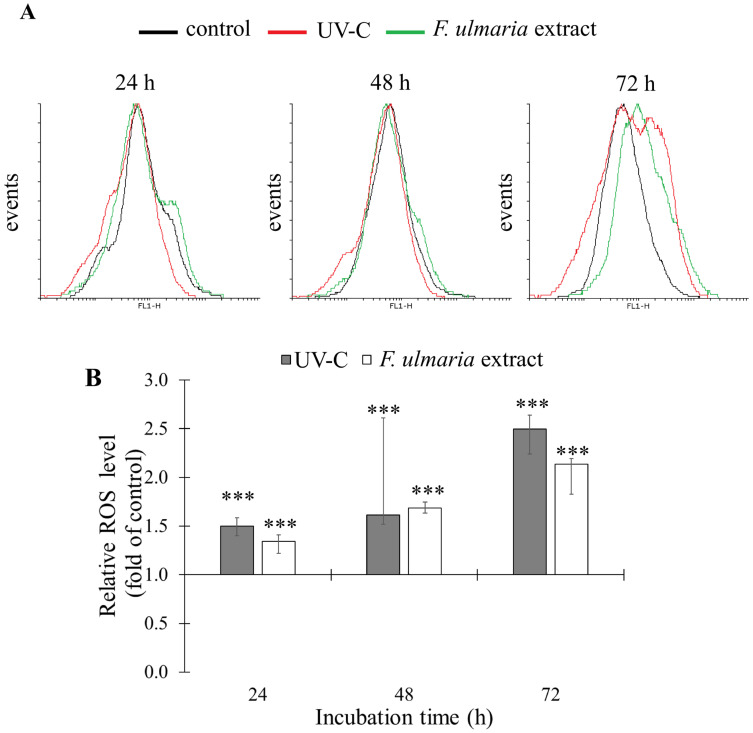
The impact of the *F. ulmaria* extract on the level of reactive oxygen species (ROS) in the LoVo cell line. (**A**) Representative histograms for cells stained with DCFH-DA, (**B**) relative ROS level in LoVo cells. The graph depicts median values and quartiles from at least three independent replicates. The level of statistical significance of differences between positive control cells (UV-C) or cells incubated with the extract compared to the negative control cell system: *** *p* ≤ 0.001. The extract was consistently applied at a definitive concentration of 0.34 mg/mL.

On a molecular level, several changes pointed to the activation of oxidative stress and apoptotic pathways in extract-treated cells. In all experimental time points, ROS levels were significantly higher than in control samples, with a peak after 72 h of treatment, when relative ROS levels in extract-exposed cells were roughly two times higher than in untreated control cells (Figure 7).

**Figure 8:**
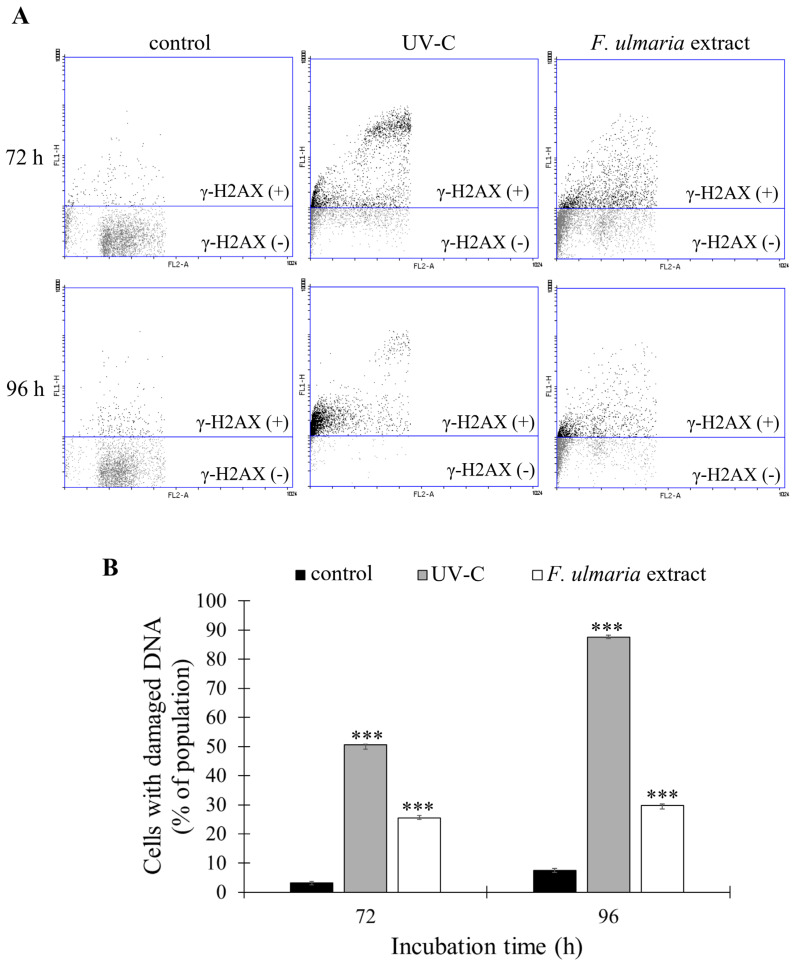
The impact of *F. ulmaria* extract on the induction of DNA damage in LoVo cell line cells. (**A**) Representative dot plots for cells stained with anti-γ-H2AX-Alexa Fluor 488 mAb and PI, (**B**) the percentage of cells with phosphorylated H2AX (γ-H2AX). The graph depicts median values and quartiles from at least three independent replicates. The level of statistical significance of differences between the cells from the positive control (UV-C) or the cells incubated with the extract, and the system of cells from the negative control: *** *p* ≤ 0.001. The extract was consistently applied at a definitive concentration of 0.34 mg/mL.

We also assessed DNA damage by measuring γ-H2AX levels, a marker of DNA double-strand breaks. The proportion of γ-H2AX-positive cells was substantially higher in extract-treated cultures compared to controls, indicating that the extract caused considerable DNA damage. The percentage of cells incubated with the extract that showed a high degree of histone H2AX phosphorylation was approximately 30% of the population, both after 72 and 96 h of the experiment (Figure 8).

**Figure 9:**
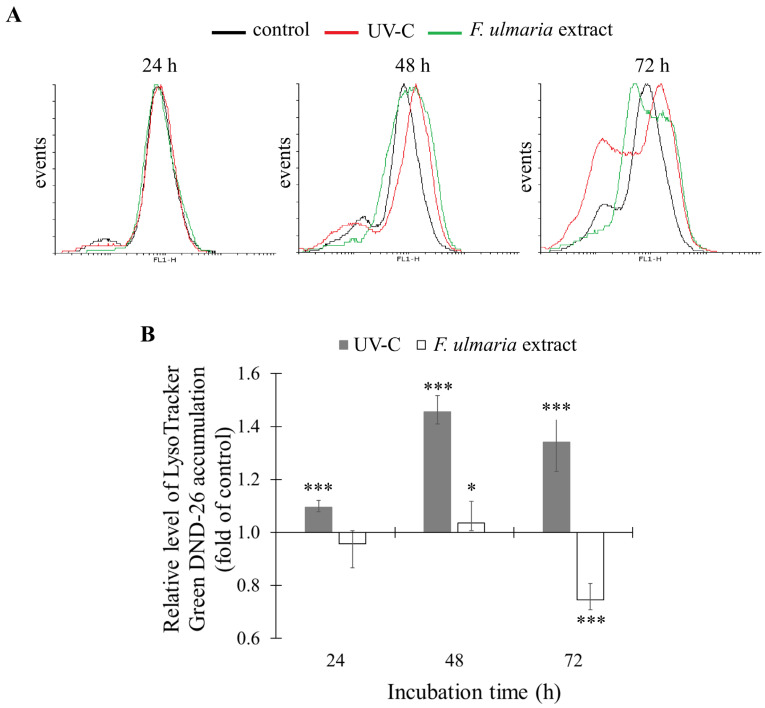
The impact of *F. ulmaria* extract on LysoTracker Green fluorescence in LoVo cells. (**A**) Representative histograms for cells stained with LysoTracker Green DND-26, (**B**) relative level of LysoTracker Green DND-26 accumulation in lysosomes expressed as a fold of control. The graphs depict median values and quartiles from at least three independent replicates. The level of statistical significance of differences between the cells from the positive control (UV-C) or the cells incubated with the extract, and the cells from the negative control: * *p* ≤ 0.05, *** *p* ≤ 0.001. The extract was consistently applied at a definitive concentration of 0.34 mg/mL.

Analysis of LysoTracker Green fluorescence indicated that the extract altered lysosomal staining characteristics. (Figure 9). A total of 24 hours and 48 hours of incubation of LoVo cells with meadowsweet extract did not cause significant changes in the accumulation of LysoTracker Green DND-26 in cell lysosomes compared to the control. However, 72 hours of incubation of LoVo cells with the extract caused a statistically significant decrease in the level of lysosomal probe accumulation. The relative fluorescence level of the tracer decreased to 0.75-fold that of the control.

**Figure 10:**
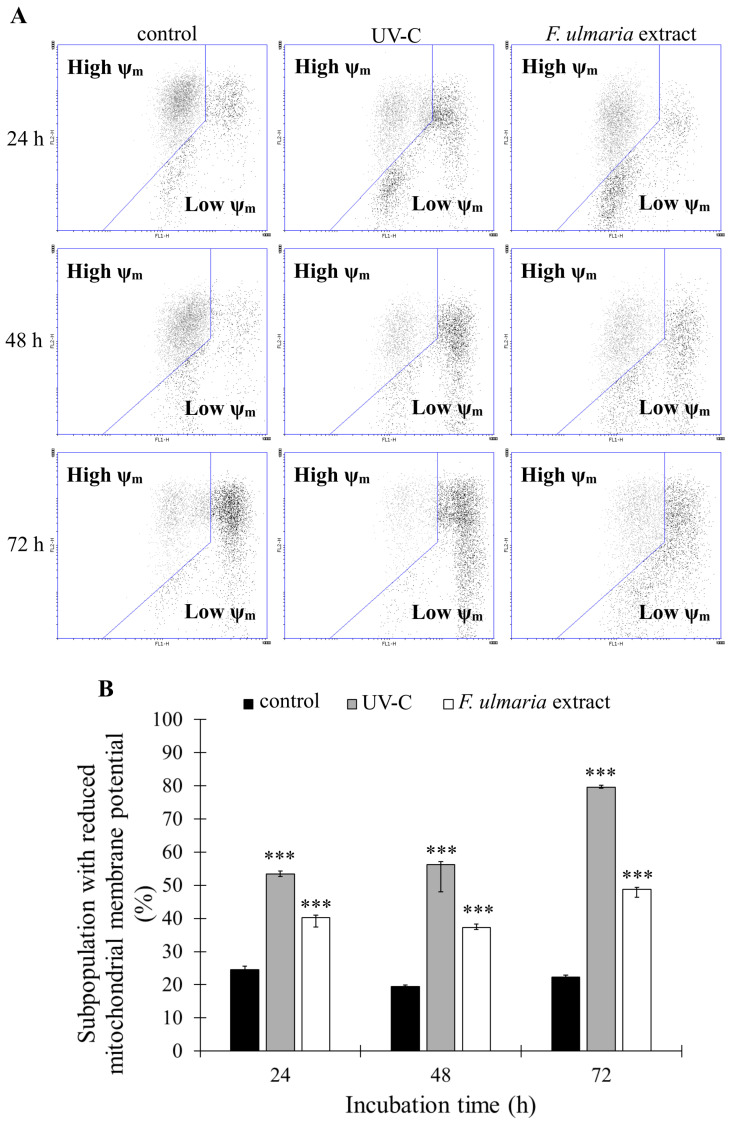
The impact of the *F. ulmaria* extract on the mitochondrial membrane potential (ΔΨm) in LoVo cell line. (**A**) Representative dot plots stained with JC-1 dye, (**B**) the percentage of cells with reduced mitochondrial membrane potential. The graph depicts median values and quartiles from at least three independent replicates. The level of statistical significance of differences between the cells from the positive control (UV-C) or the cells incubated with the extract, and the cells from the negative control: *** *p* ≤ 0.001. The extract was consistently applied at a definitive concentration of 0.34 mg/mL.

Mitochondrial function was likewise affected. Using the JC-1 dye to assess mitochondrial membrane potential (ΔΨm), we observed a clear reduction in mitochondrial membrane potential in extract-treated cells relative to controls (Figure 10). In cells incubated with meadowsweet extract, the percentage of cells with reduced ΔΨm after 24 h and 48 h of incubation was approximately 40%, and this level increased to approximately 50% after 72 h of incubation.

**Figure 11:**
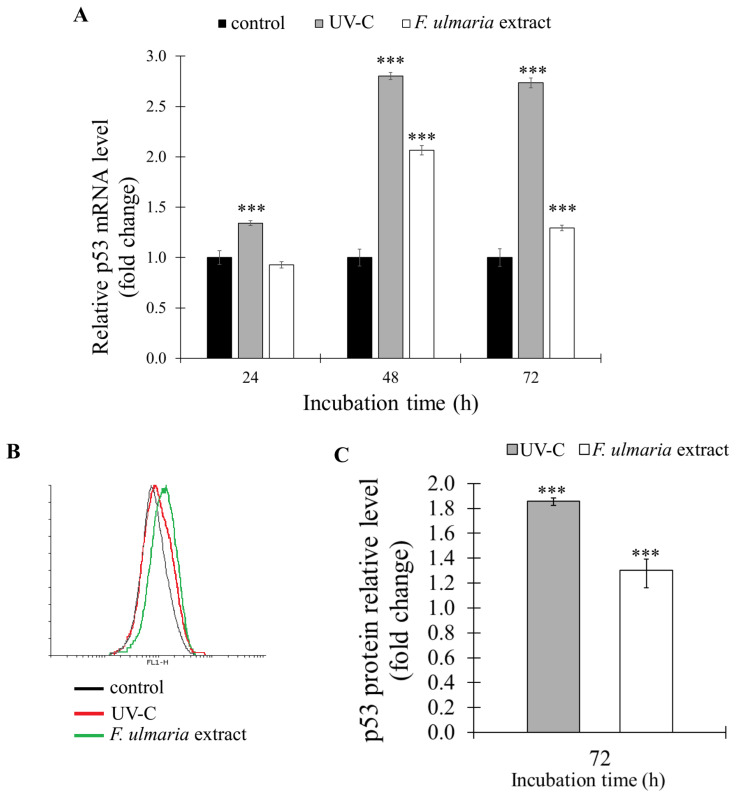
The effect of *F. ulmaria* extract on the expression level of *TP53* gene and p53 protein level in LoVo cell line. (**A**) Comparison of the expression levels of *TP53* gene, (**B**) representative histograms for cells stained with anti-p53-FITC mAb, (**C**) p53 protein relative level. The graphs depict the median from at least three independent replicates. The level of statistical significance of differences between the cells from the positive control (UV-C) or the cells incubated with the extract, and the cells from the negative control: *** *p* ≤ 0.001. The extract was consistently applied at a definitive concentration of 0.34 mg/mL.

To further elucidate the mechanisms of cell death, we examined the expression of genes and the levels of proteins involved in apoptosis induction and execution. Notable changes in the tumor suppressor protein p53, which is central to the cellular DNA damage response, were observed (Figure 11). In extract-treated LoVo cells, p53 protein levels were markedly increased relative to untreated cells. This level in cells incubated with the extract was approximately 1.3-fold higher than the control. This was accompanied by a significant rise in p53 mRNA levels.

**Figure 12:**
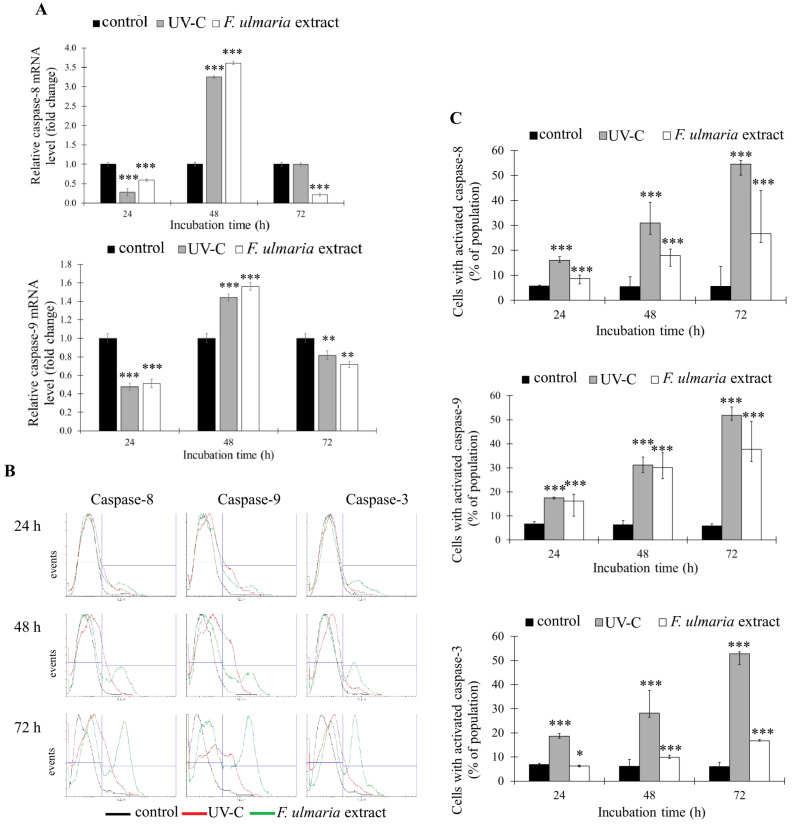
The impact of *F. ulmaria* extract on the expression of *CASP8* and *CASP9*, and the level of activated caspase-8, -9, and -3 in LoVo cell line. (**A**) Comparison of the expression levels of *CASP8 and CASP9* genes, (**B**) representative histograms for cells stained with fluorescent-labeled inhibitors of caspases, (**C**) changes in the percentage of cells with activated forms of tested caspases. The graphs depict the median from at least three independent replicates. The level of statistical significance of differences between the cells from the positive control (UV-C) or the cells incubated with extract compared to the negative control cell group: * *p* ≤ 0.05. ** *p* ≤ 0.01. *** *p* ≤ 0.001. The extract was consistently applied at a definitive concentration of 0.34 mg/mL.

Significant changes were also noted for caspases (Figure 12). Quantitative PCR analysis showed that after 48 h of extract exposure, the mRNA levels of initiator *CASP8* and *CASP9* genes were significantly upregulated (approximately 3.6-fold and 1.6-fold higher than control, respectively). No significant change in these transcripts was detected at 24 h, and by 72 h their expression levels had declined toward baseline, suggesting a transient induction of these apoptosis-related genes. While gene expression data indicated activation of apoptotic pathways, we confirmed the presence of activated caspase enzymes using the FLICA assays. In cells incubated with meadowsweet extract, the percentage of cells with active caspases after 24 h of the experiment was 8.70% (caspase-8), 16.26% (caspase-9), and 6.25% (caspase-3). After two days of exposure to the extract, these levels increased to 17.92% for caspase-8, 30.09% for caspase-9, and 9.88% for caspase-3, respectively. A 72-hour exposure of cells to the tested extract resulted in a further increase in the number of cells with active caspases. The percentage of these cells was 26.63% for caspase-8, 37.77% for caspase-9, and 16.67% for caspase-3.

**Figure 13:**
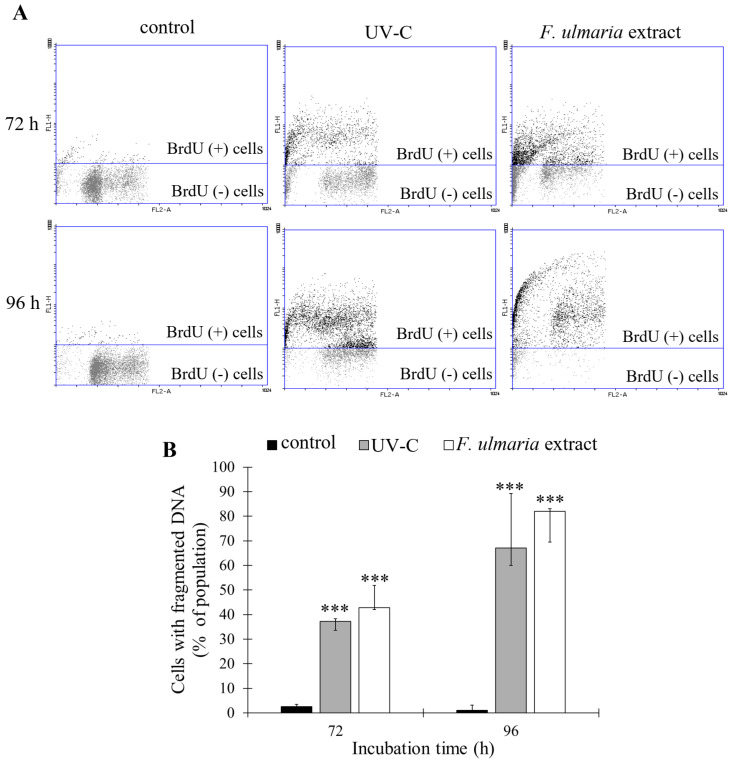
The impact of the *F. ulmaria* extract on the degree of DNA fragmentation in LoVo cell line: (**A**) representative dot plots for cells stained with anti-BrdU-FITC mAb and PI, (**B**) the percentage of cells with fragmented DNA. The graphs depict the median from at least three independent replicates. The level of statistical significance of differences between the cells from the positive control (UV-C) or the cells incubated with the extract, and the cells from the negative control: *** *p* ≤ 0.001. The extract was consistently applied at a definitive concentration of 0.34 mg/mL.

Consistent with caspase-mediated apoptosis, cells treated with the extract exhibited DNA fragmentation. The TUNEL assay results revealed that in cells incubated with meadowsweet extract, the level of DNA fragmentation after three days of the experiment was 42.83%, while after the fourth day this value increased to 82.06% (Figure 13).

## 4. Discussion

*Filipendula ulmaria* (L.) Maxim. (meadowsweet) is recognized for anti-inflammatory, antibacterial, antifungal, hepato-, nephro-, gastro-, and neuroprotective properties [9,10,11,12,13,14,15,16]. This plant belongs to the *Rosaceae* family, many of whose representatives have already been studied also for their antioxidant and anticancer potential, yet the ability of meadowsweet to induce cancer cell death had not been thoroughly investigated prior to this work. In this study, we evaluated the anticancer potential of an aqueous *F. ulmaria* extract against a human colorectal cancer cell line (LoVo). Colorectal cancer remains a leading cause of cancer-related mortality worldwide, emphasizing the need for new therapeutic approaches. *F. ulmaria.* Our study demonstrates the cytotoxic effects of a meadowsweet aqueous extract on colon cancer cells and examines some mechanisms underlying this effect.

Extracts of *F. ulmaria* are particularly rich in polyphenols and other phytochemicals. In the aqueous extract we tested, the total phenolic content was approximately 230 mg GAE/g. It is important to note that extraction solvent influences the yield and composition of polyphenols [17,18]. So far, the literature has described meadowsweet extracts prepared as aqueous, aqueous-ethanol, aqueous-acetone, and ethanol extracts. Water generally extracts fewer polyphenols than organic solvents, due to the limited solubility of many polyphenolic compounds in water [17]. Nevertheless, we chose an aqueous extraction to mimic traditional herbal preparations, since water is the most common solvent in folk usage. This approach favors the isolation of hydrophilic constituents like flavonoid glycosides and phenolic acids, which are known for antioxidant and anticancer activities. Our phytochemical analysis confirmed a diverse range of secondary metabolites in the aqueous extract of *F. ulmaria* (Figure 1). Obtained data are consistent with studies by other authors, which have shown that meadowsweet extracts retain a wide array phenolic acids (e.g., p-hydroxybenzoic acid; gallic, ellagic, syringic, coffeic, p-coumaric, vanillic, ferulic, salicylic, anisic, cinnamic), flavonoids (e.g., quercetin, miquelianin, kaempferol, astragalin, rutin, apigenin), ellagitannins (e.g., rugosins, tellimagrandins), and procyanidins [10,19,20,21,22,23]. Our analysis identified numerous bioactive compounds in the extract, with flavonoids (particularly quercetin and kaempferol glycosides) being predominant. Many of these constituents have documented anticancer properties. For example, quercetin and kaempferol derivatives can inhibit cancer cell proliferation and angiogenesis [24], and ellagic acid has pro-apoptotic and antiproliferative effects [25]. The presence of epicatechin, naringenin, coumarins like umbelliferone, and various phenolic acids (gallic, ellagic, chlorogenic, etc.) further suggests that the extract could exert broad biological effects. Each of these compound classes has been associated with health benefits and, in some cases, anticancer activities. Using an aqueous extract may also have practical advantages for colorectal applications. Hydrophilic compounds in such extracts are more likely to reach the colon intact (without being absorbed earlier in the gastrointestinal tract) compared to compounds that require lipid-based solvents for extraction. Therefore, our choice of the LoVo colon cancer cell line is particularly relevant, as it reflects the intestinal epithelial environment that an orally administered extract would encounter. Although organic solvent extracts might yield an even broader spectrum of phytochemicals, our findings show that a simple water extract still contains sufficient active compounds to induce strong biological effects. Future studies could compare different extraction solvents (water vs. aqueous alcohols vs. pure organics) [26] to optimize the composition and efficacy of meadowsweet extracts.

In line with its rich phytochemical profile, the aqueous meadowsweet extract demonstrated significant antioxidant activity in chemical tests. Previously, meadowsweet extracts have been shown to inhibit the growth of cancer cells (e.g., HepG2 liver cancer) while exhibiting antioxidant effects [27]. Our results reinforce the idea that *F. ulmaria* could be useful in cancer prevention or therapy, given its capacity to scavenge free radicals and reduce oxidants. The meadowsweet extract displayed robust antioxidant capacity in all of the chemical assays we employed. In reducing power assays (FRAP and TAC), the extract was able to neutralize 80–90% of the oxidant challenge, which is remarkably high and in agreement with previous findings. For instance, Neagu et al. (2015) reported that *F. ulmaria* extracts exhibited antioxidant activities comparable to or exceeding that of ascorbic acid [23]. Similarly, Samardžić et al. (2018) noted substantial reducing activity in meadowsweet flower extracts, albeit slightly lower than ascorbic acid [10]. Other *Rosaceae* extracts, such as those from wild apple and plum, have also shown high FRAP values [28,29], underscoring that plants in this family often have strong antioxidant properties. In the DPPH assay, our extract scavenged nearly 90% of DPPH radicals, essentially matching the efficacy of ascorbic acid (this mirrors earlier observations by other authors [10,23,30]). In the superoxide radical assay, the extract actually outperformed ascorbic acid in quenching superoxide (O_2_^•−^). This superior activity is consistent with the work of Trouillas et al. (2003), who found that meadowsweet had the highest superoxide scavenging capacity among 16 tested plant extracts [31]. The ABTS scavenging results further confirmed the extract’s potency, with performance on par with standard antioxidants [32]. Overall, our antioxidant findings are in line with numerous reports that *F. ulmaria* and related *Filipendula* species have strong radical-scavenging and reducing capabilities [33,34].

Using the MTT assay, we demonstrated that the aqueous *F. ulmaria* extract significantly reduces the viability of LoVo colorectal cancer cells (IC_50_ ≈ 0.34 mg/mL after 72 h). The tested meadowsweet extract also demonstrated low activity against normal BJ fibroblast cells. The IC_50_ value for these cells was 41-fold higher than for LoVo cells. This difference indicates a clearly selective effect of the tested extract on cancer cells and can be interpreted as a positive indicator of the extract’s therapeutic potential. Notably, this is the first study to report a cytotoxic effect of *F. ulmaria* extract on human colon cancer cells [14]. We chose the LoVo cell line as our model because it is commonly used in studies of plant-derived compounds with gastrointestinal relevance. LoVo cells display key characteristics of colorectal tumors, including oxidative stress imbalance and high susceptibility to pro-apoptotic stimuli, making them a suitable in vitro system for our investigation.

From a therapeutic standpoint, targeting colorectal cancer with plant extracts is appealing because oral administration allows direct contact between bioactive compounds and the intestinal epithelium. The anticancer potential of *F. ulmaria* and similar preparations has been noted in earlier studies involving other cancer models. For example, methanolic meadowsweet extracts tested via the XTT assay showed IC_50_ values greater than 50 µg/mL in glioblastoma (U-251) and colon cancer (HCT 116) cells [35]. Infusions from meadowsweet flowers achieved approximately 50% growth inhibition in non-small cell lung cancer (NCI-H460) at ~70 µg/mL, in breast cancer (MCF-7) at ~96 µg/mL, and in melanoma (A375-C5) at ~63 µg/mL [8]. Similarly, methanolic extracts from *F. ulmaria* rhizomes were cytotoxic to U-251 and HCT 116 cells with IC_50_ values above 50 µg/mL [33]. Compared to those findings, our aqueous extract required higher concentrations (hundreds of µg/mL) to reach IC_50_ in LoVo cells, which is reasonable given the differences in extract type and cell line. These comparisons suggest that while meadowsweet contains cytotoxic agents, the potency can vary with extraction method and cancer cell type.

Considering the extract’s strong antioxidant capacity in chemical assays, it was pertinent to examine its effect on intracellular ROS in cancer cells. At first glance, the increase in ROS observed in LoVo cells may appear inconsistent with the radical-scavenging activity demonstrated in the DPPH and ABTS assays. However, these assays evaluate direct chemical interactions between antioxidants and free radicals under cell-free conditions and do not reflect the complexity of the intracellular environment. ROS play complex roles in cancer biology: most tumors exhibit elevated ROS levels, but cancer cells adapt by upregulating antioxidant defenses. This balance between pro-oxidants and antioxidants influences tumor progression and can affect how cancer cells respond to therapy. Plant-derived compounds are known to act as antioxidants in normal cells but can paradoxically act as pro-oxidants in cancer cells, exploiting the cancer cells’ already strained redox homeostasis. Such dual activity can lead to pleiotropic effects on multiple molecular pathways. While polyphenols efficiently scavenge free radicals in chemical assays through electron or hydrogen donation, under specific intracellular conditions they may also promote ROS generation in cancer cells [36,37,38,39]. One possible explanation is that polyphenolic constituents of the extract undergo redox cycling or transition-metal-catalyzed oxidation in cancer cells, leading to increased production of reactive oxygen species. Because cancer cells already operate close to the limit of their redox buffering capacity, even a moderate increase in ROS may disrupt cellular homeostasis and activate oxidative stress-related signaling pathways. Our data indicate that the meadowsweet extract drives a pro-oxidant effect in LoVo cells. After 72 h of treatment, ROS levels roughly doubled in treated cells compared to untreated cells. By disrupting the cellular redox equilibrium, the extract likely inflicts oxidative damage to crucial biomolecules (DNA, lipids, proteins), thereby activating signaling pathways that lead to cell cycle arrest or apoptosis. In other words, part of the extract’s anticancer activity may stem from its ability to induce oxidative stress beyond what the cancer cells can tolerate. To our knowledge, there have been no previous reports specifically on *F. ulmaria* extracts raising ROS in cancer cells. However, related observations exist for other plant products: for instance, an emulsion of raspberry seed oil (another *Rosaceae* plant) significantly increased ROS levels in LoVo and MCF-7 cells [40]. In contrast, an aqueous *Vaccinium meridionale* (Andean blueberry) extract did not affect ROS in SW480 and SW620 colon cancer cells [41]. Thus, our findings shed new light on meadowsweet, revealing that it can act as a pro-oxidant in cancer cells. This opens up questions about the molecular basis of this effect. Future studies should investigate how the extract influences cellular antioxidant systems (e.g., activities of enzymes like superoxide dismutase, catalase, glutathione peroxidase), the status of thiol antioxidants (glutathione, thioredoxin), and other redox-regulated pathways. Understanding these details will clarify how oxidative stress contributes to the extract’s cytotoxicity.

We also explored whether the extract affects lysosomal status. Lysosomes are key mediators of cell death when they become destabilized: leakage of lysosomal enzymes into the cytosol can trigger apoptosis or necrosis. Lysosomes are particularly sensitive to oxidative stress, so an increase in ROS levels may affect lysosomal properties [42,43,44]. In our study, for the first time, we demonstrated that *F. ulmaria* extract treatment significantly reduced LysoTracker Green accumulation in LoVo cells. This finding suggests alterations in lysosomal status; however, changes in LysoTracker fluorescence alone are insufficient to conclusively demonstrate lysosomal dysfunction or lysosomal membrane permeabilization. Therefore, the present findings should be interpreted as evidence of altered lysosomal properties rather than direct proof of LMP. Lysosomal involvement in cell death has been reported for many natural compounds. Resveratrol, for example, induces LMP in cervical carcinoma cells [45]; epigallocatechin gallate (EGCG) from green tea triggers lysosomal damage in HepG2 liver cancer cells [46]; and saponin tubeimoside-1 causes LMP in NCI-H1299 and NCI-H1975 non-small cell lung cancer cells [47]. Additionally, an extract from *Pinus radiata* was shown to destabilize lysosomes in MCF-7 breast cancer cells [48]. Our findings with meadowsweet extract are in line with previous reports indicating that plant-derived compounds can alter lysosomal properties. Additional studies will be required to determine the nature of the observed lysosomal alterations and their contribution to the extract-induced cytotoxicity. Although the meadowsweet extract did not cause a classic cell cycle arrest at G_1_/S or G_2_/M checkpoints in LoVo cells, it clearly disrupted normal cell cycle progression. The transient G_1_ phase accumulation we observed at early time points could reflect a stress response (cells pausing in G_1_). With longer exposure, the drop in G_1_ cells and concomitant rise in sub-G_1_ events indicate that many cells did not progress through the cycle but instead underwent apoptosis. The Annexin V/PI assay confirmed apoptosis as the main form of cell death under extract treatment. These findings suggest that the extract’s cytotoxicity is not due to halting the cell cycle at a specific phase (as some treatments do), but rather due to pushing cells out of the cycle into apoptosis.

Studies on other *Rosaceae* plant extracts have reported varying effects on the cancer cell cycle. Lima et al. (2014) found that a meadowsweet flower decoction had minimal impact on cell cycle distribution in NCI-H460 lung cancer cells [8]. Conversely, an ethyl acetate extract from hawthorn (*Crataegus azarolus*) leaves caused a modest G_1_ arrest and an increase in sub-G_1_ in HT-29 colon cancer cells, as well as similar effects in HCT 116 cells [49]. Another study showed that a methanolic *Prunus spinosa* seed extract, combined with a nutraceutical apoptosis sensitizer, led to cell cycle shifts (increased sub-G_1_ and altered G_1_/S ratios) in HCT 116 and SW480 colon cancer cells [50]. These examples highlight that plant extracts can influence the cell cycle in diverse ways, often linked with their ability to induce apoptosis [51,52]. In agreement with those observations, our results demonstrate the pro-apoptotic potential of *F. ulmaria* extract in a colorectal cancer model. This is further supported by similar reports: for example, a methanolic extract of bee pollen rich in *F. ulmaria* content induced apoptosis in C26 murine colon carcinoma cells [53], and an ethanolic extract from *Rubus sanctus* (another *Rosaceae* plant) triggered apoptosis in LoVo and HT-29 colon cancer cells [54]. Likewise, an aqueous *Vaccinium meridionale* fruit extract caused apoptosis in SW480 and SW620 cells [38], and *Prunus mume* (Japanese apricot) extract induced apoptosis in SW480, COLO 205, and WiDr colon cancer cell lines [55]. Taken together, these findings underscore that many *Rosaceae* extracts, including *F. ulmaria*, harbor components capable of driving apoptosis in colorectal cancer cells.

A key feature of apoptosis is the loss of mitochondrial membrane potential, which is often an early event in the intrinsic pathway of apoptosis [56]. Our JC-1 assay results showed that the extract causes mitochondrial depolarization in LoVo cells, confirming the involvement of the intrinsic pathway. This mechanism is consistent with other studies on *Rosaceae* extracts. For instance, Mustapha et al. (2016) reported that an ethyl acetate hawthorn extract dissipated ΔΨm in HT-29 and HCT 116 cells, leading to apoptosis [49]. Similarly, a root extract of *Sanguisorba officinalis* (another member of *Rosaceae*) reduced ΔΨm in HCT 116 cells (although interestingly it did not affect ΔΨm in RKO colon cancer cells, suggesting cell line–specific responses) [57]. Our findings align with these, indicating that mitochondrial disruption is part of how the meadowsweet extract induces cell death.

At the gene expression level, we observed transient activation of the apoptosis-related genes *CASP8* and *CASP9*. The upregulation at 48 h suggests that the extract quickly initiates apoptotic signaling, but by 72 h, transcript levels declined, possibly because many cells had already undergone apoptosis or because of feedback regulation. It is notable that the degree of caspase gene induction in extract-treated cells was similar to that caused by UV-C radiation (a known strong inducer of apoptosis), highlighting the extract’s potency. However, mRNA upregulation alone does not guarantee that caspases are active, since the translation and post-translational activation steps are critical. Indeed, prior research cautions that increases in procaspase levels might not directly reflect active caspase levels [58]. For this reason, we corroborated our RT-qPCR results with functional assays for caspase activity. Using FLICA probes, we directly confirmed that caspase-8, caspase-9, and caspase-3 become activated in response to the extract. Caspase-9 is a key executor of the intrinsic (mitochondrial) apoptotic pathway, while caspase-8 is an initiator of the extrinsic (death receptor) pathway. The simultaneous activation of both caspases strongly suggests that the extract triggers a dual apoptotic mechanism: it damages mitochondria (intrinsic route) and possibly engages death receptors on the cell surface (extrinsic route). The involvement of both pathways could be due to extensive crosstalk once apoptosis is underway (for example, caspase-8 can cleave Bid to amplify mitochondrial apoptosis). Our results are in agreement with other findings in the literature. An ethanolic extract of *Rubus sanctus* roots was shown to increase the proportion of cells with active caspases in colon cancer lines [54]. Additionally, *Prunus mume* fruit extract activated caspases-8, -9, and -3 in SW480 colon cells, and *Sanguisorba officinalis* extract activated caspases in HCT 116 and RKO cells [57,59,60]. Thus, caspase-dependent apoptosis appears to be a common outcome of many *Rosaceae* plant extracts in colorectal cancer models, with our meadowsweet extract being no exception.

The concurrent activation of caspase-8 and caspase-9 by the extract indicates that it engages both major apoptotic pathways. This likely results from upstream events such as oxidative mitochondrial damage (triggering the intrinsic pathway) and perhaps the activation of death receptors or extrinsic signals (triggering caspase-8). Elucidating these upstream triggers will require further investigation. It would be informative to determine whether the extract influences death receptor pathways like Fas/FasL or TNF-related apoptosis-inducing ligand (TRAIL) receptors, as well as how it affects Bcl-2 family proteins that regulate mitochondrial apoptosis.

While direct evidence of extrinsic pathway activation by meadowsweet is not yet available, the pattern we observed (caspase-8 activation, rapid apoptosis) is reminiscent of what has been seen with other plant extracts. As mentioned, *Rubus sanctus* root extract and *Prunus mume* extract both activate multiple caspases in colon cancer cells [54,57]. These parallels suggest that *F. ulmaria* shares a mode of action with those extracts, potentially involving cell-surface death receptors in addition to internal damage signals.

Apoptosis induced by severe cellular stress is often accompanied by DNA fragmentation. In our study, the TUNEL assay confirmed extensive DNA fragmentation in extract-treated cells, aligning with the late-stage apoptosis indicated by sub-G_1_ accumulation. DNA fragmentation typically results from endonuclease activation downstream of caspases (e.g., CAD nuclease activated by caspase-3) [61]. The presence of fragmented DNA underscores that the extract pushes cells beyond repair.

One major trigger for apoptosis is irreparable DNA damage. Cancer cells endure high levels of DNA damage but survive by invoking robust DNA repair mechanisms [62]. The balance between DNA damage and repair is partly overseen by the tumor suppressor p53, often called the “guardian of the genome” [63]. When DNA damage (such as double-strand breaks) accumulates, p53 is stabilized and activated to either halt the cell cycle and facilitate repair or to initiate apoptosis if the damage is too great.

We found that the meadowsweet extract caused substantial DNA damage in LoVo cells, evidenced by a large increase in γ-H2AX–positive cells (Figure 8). H2AX phosphorylation is an early step in the DNA damage response to double-strand breaks. Interestingly, a previous study reported only a slight and statistically non-significant increase in γ-H2AX in NCI-H460 lung cancer cells treated with a meadowsweet decoction [8]. In contrast, our extract induced a pronounced γ-H2AX response in LoVo cells. This difference could be due to cell type (colon vs. lung), extract preparation (our extract might have a stronger pro-oxidant component), or exposure conditions. The strong γ-H2AX signal in our experiments likely reflects the high ROS environment generated by the extract, which can directly cause double-strand breaks [64].

Consistent with the extensive DNA damage and ongoing oxidative stress, we observed significant activation of p53 in extract-treated cells. Both p53 mRNA and protein levels were elevated (Figure 11). This indicates that the cells activated the p53 pathway in response to the extract. Notably, Lima et al. did not find a p53 protein increase in meadowsweet-treated NCI-H460 cells [8], which might mean the stress imposed by their treatment was insufficient to engage p53, or those cells responded differently. However, other studies support our findings: in HCT 116 colon cancer cells, oligomeric proanthocyanidins and hawthorn extracts induced cell death in a p53-dependent manner [49,60].

In our study, the upregulation of p53 is most likely a direct consequence of DNA damage under oxidative stress. p53 then contributes to the extract’s cytotoxic effect by imposing cell cycle arrest (we did see a transient G_1_ arrest) and promoting apoptosis [65]. The sequence of events we observed: ROS surge, DNA damage, p53 activation, apoptosis, highlights a plausible pathway: the extract causes oxidative DNA damage, which activates p53, leading to growth arrest and the initiation of apoptosis. The eventual decline in G_1_-phase cells and large increase in sub-G_1_ (apoptotic) population in treated cells is consistent with p53 driving cells out of the cycle and into programmed death.

Similar p53-related outcomes have been documented with other plant extracts. For example, an extract from *Emilia sonchifolia* induced oxidative stress and p53-mediated apoptosis in HCT 116 cells [66], and an *Inula viscosa* extract caused p53-associated cell death in MC38 murine colon carcinoma cells [67]. These parallels further support the interpretation that *F. ulmaria* extract kills LoVo cells through oxidative DNA damage and activation of a p53-dependent apoptotic program.

While this study provides novel insights into the antioxidant and anticancer effects of *F. ulmaria* extract, it is important to acknowledge its limitations. Firstly, we used an aqueous extract to reflect traditional usage and to favor compounds that might reach the colon. However, water extraction yields a narrower chemical profile compared to organic solvents. Some hydrophobic yet potentially potent phytochemicals could be underrepresented in our extract [17]. Future work should compare aqueous extracts with alcohol or other solvent extracts to determine if a broader range of compounds enhances anticancer efficacy or reveals different mechanisms. Furthermore, while our HPLC-TOF/MS analysis revealed a rich phytochemical profile comprising flavonoids, phenolic acids, terpenoids, quinones, and coumarins, the specific constituents responsible for the observed cytotoxic activity remain to be identified. It is currently unclear whether the anticancer effects arise from one or a few dominant compounds or from synergistic interactions among multiple metabolites. Future bioactivity-guided fractionation studies coupled with biological evaluation of isolated compounds will be necessary to clarify their individual and combined contributions to the observed effects.

Secondly, although the study included both LoVo colorectal cancer cells and BJ normal fibroblasts, the anticancer activity was evaluated in only one colorectal cancer cell model. While LoVo cells are a well-established model of colorectal carcinoma and BJ fibroblasts provided a preliminary assessment of selectivity toward non-malignant cells, the findings cannot yet be generalized across the molecular heterogeneity of colorectal cancer. Additional studies on multiple colorectal cancer cell lines (such as HCT 116, HT-29, SW480) would help verify that the cytotoxic effects of the extract are consistent across genetic backgrounds. Moreover, testing the extract on non-cancerous colon epithelial cells would be valuable to assess its selectivity and potential toxicity to normal tissues.

Thirdly, this research was limited to an in vitro context. Cell culture models allow us to dissect cellular mechanisms, but they cannot replicate the complexity of a living organism. Factors like bioavailability, metabolism of extract components, and systemic toxicity remain unknown. Additionally, although the extract induced ROS generation, p53 upregulation, caspase activation, and apoptosis, the causal relationships among these events have not yet been fully established. Future studies employing pharmacological inhibition or genetic silencing approaches will be required to determine the relative contribution of oxidative stress, p53 signaling, and caspase activation to the overall cytotoxic response. Therefore, in vivo studies are a crucial next step. Experiments in animal models of colorectal cancer (for example, mice with xenograft or carcinogen-induced tumors) should be conducted to evaluate whether the extract can impede tumor growth, how it is distributed and metabolized in the body, and whether any adverse effects occur at effective doses.

In summary, our findings lay a foundation for exploring *F. ulmaria* as a plant-derived adjunct in colorectal cancer therapy. The extract’s ability to induce oxidative stress and apoptosis in cancer cells, combined with its inherent antioxidant components, makes it an intriguing candidate for further development. Future research should focus on optimizing the extraction method to maximize anticancer constituents, identifying the key bioactive compounds responsible for the observed activity, elucidating the mechanistic contribution of oxidative stress- and apoptosis-related pathways, testing the extract across a broader range of cancer models, and performing preclinical animal studies. Such investigations will help determine the practical potential of meadowsweet extract in cancer prevention or treatment and guide any necessary modifications (e.g., formulation improvements or combination with other therapies) for clinical application.

## 5. Conclusions

This study demonstrates that an aqueous extract of *Filipendula ulmaria* exerts multifaceted anticancer effects on colorectal cancer cells. The extract’s cytotoxicity is driven by a combination of oxidative stress induction, mitochondrial and lysosomal dysfunction, DNA damage, and activation of both intrinsic and extrinsic apoptotic pathways. At the same time, the extract retains strong antioxidant capacity in cell-free assays, highlighting its complex biochemical profile. These results support the further investigation of *F. ulmaria* as a potential complementary agent in colorectal cancer therapy. To advance toward clinical relevance, future studies should explore different extraction techniques to enrich active compounds, evaluate the extract’s efficacy and safety in animal models, and ultimately determine whether its promising in vitro effects can translate into therapeutic benefits in vivo.

## Data Availability

Data presented in this study is contained within the article and Supplementary Material. Further inquiries can be directed to the corresponding authors.

## Supplementary Materials

The following supporting information can be downloaded at: https://www.mdpi.com/article/10.3390/biomedicines14071551/s1, Table S1: Secondary metabolites identified in the aqueous extract of *Filipendula ulmaria via* HPLC-TOF/MS; Table S2: Assessment of the Antioxidant Activity of the Extract.
