# Supplementary material for: Preliminary Assessment of Anticancer Activity of Aqueous Meadowsweet (Filipendula ulmaria (L.) Maxim.) Extract in LoVo Colorectal Cancer Cells
Source: Biomedicines. 2026 Jul 10;14(7):1551. doi: 10.3390/biomedicines14071551 (PMC13404949; doi:10.3390/biomedicines14071551)
Supplement: Supplementary file 1 [file biomedicines-14-01551-s001.zip › Table S1 Biomedicines_2026_Sobczak et al_proofread.pdf]

Table S1. Secondary metabolites identified in the aqueous extract of *Filipendula ulmaria via* HPLC-TOF/MS.

Ontology	No.	m/z	Reference m/z	Δm	rt	Ionization type	Formula	EIC chromatogram	Q3 chromatogram	Metabolite name
Flavonoids	1	447.10031	447.09329	15.70	19.98	[M-H]-	$C_{21}H_{20}O_{11}$	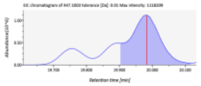	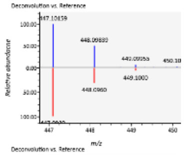	Orientin
	2	303.05202	303.05063	4.59	20.05	[M+H]+	$C_{15}H_{10}O_{7}$	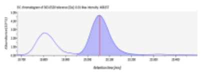	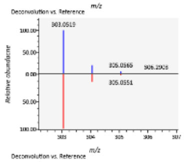	Quercetin
	3	611.16381	611.16138	3.98	19.05	[M+H]+	$C_{27}H_{30}O_{16}$	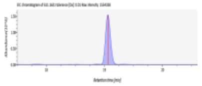	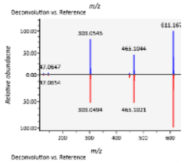	Rutin
	4	595.16691	595.16602	1.50	19.48	[M+H]+	$C_{27}H_{30}O_{15}$	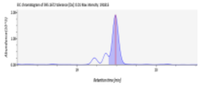	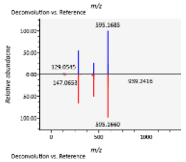	Nicotiflorin
	5	593.15325	593.15118	3.49	19.43	[M-H]-	$C_{27}H_{30}O_{15}$	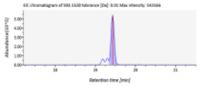	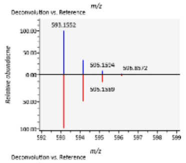	Nicotiflorin
	6	301.03737	301.03537	6.64	22.27	[M-H]-	$C_{15}H_{10}O_{7}$	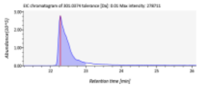	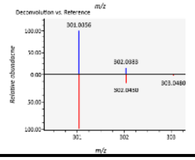	Tricetin

7	273.07692	273.07681	0.40	19.42	[M-H]-	$C_{15}H_{14}O_5$	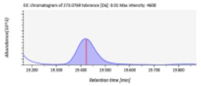	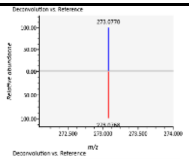	Phloretin
8	291.08739	291.08701	1.31	18.59	[M+H]+	$C_{15}H_{14}O_6$		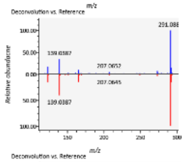	(-)-Epicatechin
9	479.12149	479.12000	3.11	19.75	[M+H]+	$C_{22}H_{22}O_{12}$	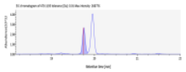	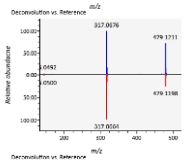	Isorhamnetin-3-glucoside
10	477.07187	477.07101	1.80	18.34	[M-H]-	$C_{21}H_{18}O_{13}$	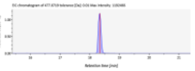	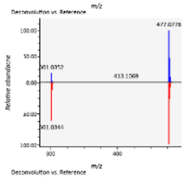	Quercetin-3-o-b-d-glucuronide
11	465.10642	465.10300	7.35	20.12	[M+H]+	$C_{21}H_{20}O_{12}$	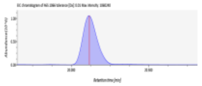	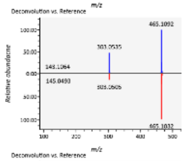	Spiraeoside
12	465.10427	465.10345	1.76	19.46	[M+H]+	$C_{21}H_{20}O_{12}$	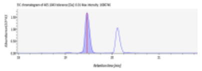	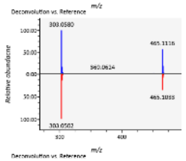	Hyperoside
13	463.10054	463.08820	26.65	20.04	[M-H]-	$C_{21}H_{20}O_{12}$	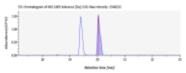	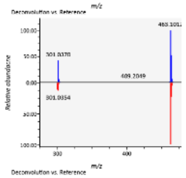	Isoquercitrin
14	463.08858	463.08801	1.23	19.99	[M+H]+	$C_{21}H_{18}O_{12}$	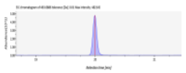	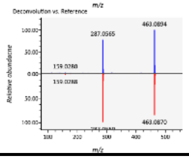	Kaempferol 3-glucuronide

15	449.09311	449.10779	32.69	4.94	[M+H]+	$C_{21}H_{20}O_{11}$	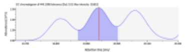	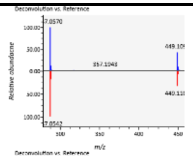	Luteolin 4'-O-glucoside
16	447.10774	447.09341	32.05	20.21	[M-H]-	$C_{21}H_{20}O_{11}$	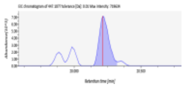	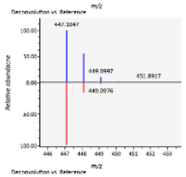	Kaempferol-3-O-glucoside
17	623.16442	623.16174	4.30	10.03	[M-H]-	$C_{28}H_{32}O_{16}$	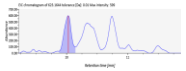	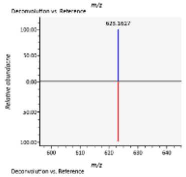	Isorhamnetin-3-O-galactoside-6'-rhamnoside
18	419.09848	419.09726	2.91	20.41	[M]+	$C_{20}H_{19}O_{10}$	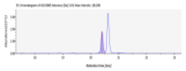	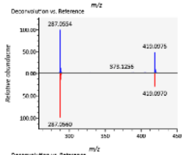	Cyanidin-3-O-alpha-arabinoside
19	417.08825	417.08273	13.23	20.56	[M-H]-	$C_{20}H_{18}O_{10}$	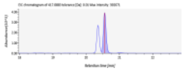	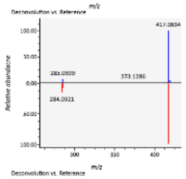	Kaempferol-3-O-arabinoside
20	287.05438	287.05502	2.23	19.44	[M+H]+	$C_{15}H_{10}O_{6}$	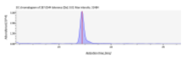	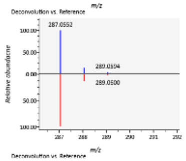	Fisetin
21	271.06516	271.06119	14.65	23.16	[M-H]-	$C_{15}H_{12}O_{5}$	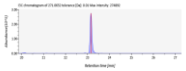	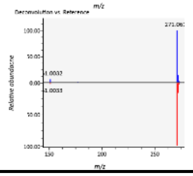	Naringenin

	22	582.21965	582.21997	0.55	16.5	[M+H]+	$C_{33}H_{31}N_{3}O_{7}$	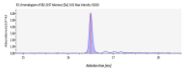	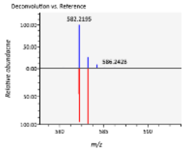	5,7-dihydroxy-8-(1-(2-hydroxy-6-methoxyquinolin-3-yl)-3-(4-methylpiperazin-1-yl)-3-oxopropyl)-2-phenyl-4H-chromen-4-one
Phenolic acids	23	300.99928	300.99899	0.96	17.92	[M-H]-	$C_{14}H_{6}O_{8}$	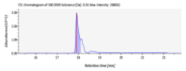	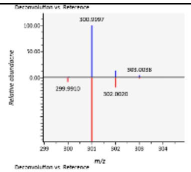	Ellagic Acid
	24	353.0888	353.08780	2.83	15.36	[M-H]-	$C_{16}H_{18}O$	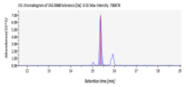	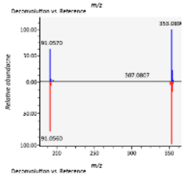	Chlorogenic acid
	25	137.02469	137.02441	2.04	12.94	[M-H]-	$C_{7}H_{6}O_{3}$	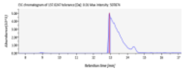	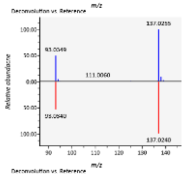	3-hydroxybenzoic acid
	26	153.01977	153.01933	2.88	17.16	[M-H]-	$C_{7}H_{6}O_{4}$	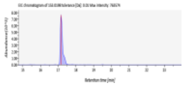	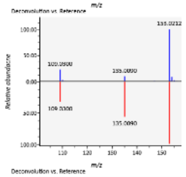	2_6_dihydroxybenzoic_acid
	27	181.04931	181.05000	3.81	17.36	[M+H]+	$C_{9}H_{8}O_{4}$	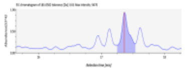	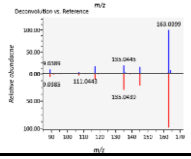	Caffeic Acid

	28	163.0404	163.04007	2.02	16.91	[M-H]-	$C_9H_8O_3$	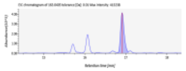	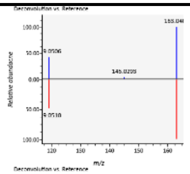	p-Coumaric acid
	29	169.01498	169.01425	4.32	2.43	[M-H]-	$C_7H_6O_5$	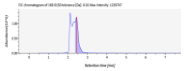	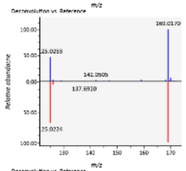	Gallic acid
	30	163.03924	163.03799	7.67	17.23	M-H2O+H	$C_9H_8O_4$	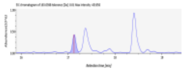	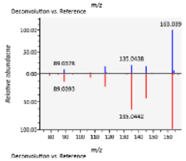	Caffeic Acid
	31	153.01968	153.01933	2.29	2.96	[M-H]-	$C_7H_6O_4$	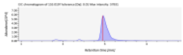	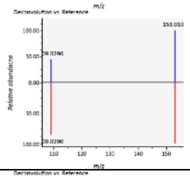	Protocatechic Acid
Coumarins	31	161.02432	161.02441	0.56	15.94	[M-H]-	$C_9H_6O_3$	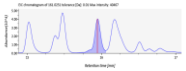	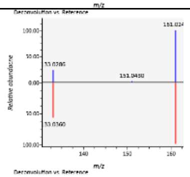	Umbelliferone (7-Hydroxycoumarin)
	32	177.01931	177.01930	0.06	18.66	[M-H]-	$C_9H_6O_4$	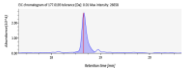	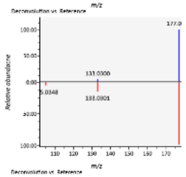	Esculetin (6,7-Dihydroxycoumarin)
	33	271.06085	271.06100	0.55	22.88	[M-H]-	$C_{15}H_{10}O_5$	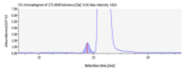	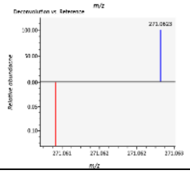	Thunberginol A

Terpenoids	34	267.07406	267.07349	2.13	14.58	[M-H]-	$C_{10}H_{12}N_4O_5$	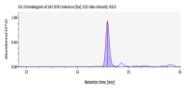	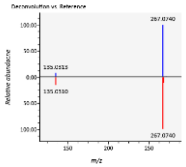	3,12-dihydroxy-4,6a,6b,11,12,14b-hexamethyl-1,2,3,4a,5,6,7,8,9,10,11,12a,14,14a-tetradecahydropicene-4,8a-dicarboxylic acid
	35	503.33689	503.33719	0.60	22.79	[M-H]-	$C_{30}H_{48}O_6$	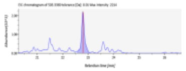	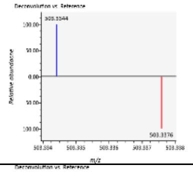	Madecassic acid
Quinones	36	271.02406	271.02481	2.77	19.97	[M-H]-	$C_{14}H_8O_6$	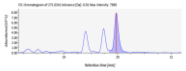	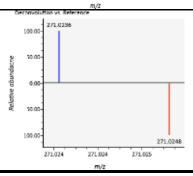	Quinalizarin

Note: Nicotiflorin was detected in both positive ([M+H]$^+$) and negative ([M–H]$^-$) ionization modes and therefore appears twice (compounds 4 and 5).
