# Supplementary material for: Preliminary Assessment of Anticancer Activity of Aqueous Meadowsweet (Filipendula ulmaria (L.) Maxim.) Extract in LoVo Colorectal Cancer Cells
Source: Biomedicines. 2026 Jul 10;14(7):1551. doi: 10.3390/biomedicines14071551 (PMC13404949; doi:10.3390/biomedicines14071551)
Supplement: Supplementary file 1 [file biomedicines-14-01551-s001.zip › Table S2 Biomedicines_2026_Sobczak et al_proofread.pdf]

Table S2. Assessment of the Antioxidant Activity of the Extract.

	Relative Reducing Power (%)				Radicals Scavenging Activity (%)					
	**FRAP**		**TAC**		**DPPH**		**ABTS$^{\bullet+}$**		**$O_2^{\bullet-}$**	
**Concentration (mg/mL)**	Ascorbic acid	Extract	Ascorbic acid	Extract	Ascorbic acid	Extract	Ascorbic acid	Extract	Ascorbic acid	Extract
0.01									8.89	6.62
0.05	31.45	10.61					27.82	13.10		
0.1	64.31	47.32	95.06	83.22	35.86	23.12	56.67	30.03	6.79	21.48
0.25	77.34	52.55	96.40	93.22			94.23	61.56		
0.5			98.69	95.05	35.66	36.14	94.33	91.54	4.50	64.60
0.75	92.11	86.92	99.14	95.94						
1			99.55	96.35	32.35	30.92	94.27	94.33	18.55	81.99
2	93.03	91.86	99.52	98.00						
10					67.57	57.71			15.32	90.40
50					79.60	85.32				

Table summarizes data obtained in Ferric Ion Reducing Antioxidant Power (FRAP) assay, Total Antioxidant Capacity (TAC) - molybdenum blue method, 2,2-diphenyl-1-picrylhydrazyl (DPPH) radical scavenging assay, 2,2′-azino-bis(3-ethylbenzothiazoline-6-sulfonic acid) (ABTS) radical cation decolorization assay, and superoxide anion radical scavenging assay.
